# 2-Hydroxypropyl-β-cyclodextrin (HPβCD) as a Potential Therapeutic Agent for Breast Cancer

**DOI:** 10.3390/cancers15102828

**Published:** 2023-05-18

**Authors:** Sourav Taru Saha, Naaziyah Abdulla, Tawanda Zininga, Addmore Shonhai, Reubina Wadee, Mandeep Kaur

**Affiliations:** 1 School of Molecular and Cell Biology, University of the Witwatersrand, Private Bag 3, WITS-2050, Johannesburg 2050, South Africa; 2 Department of Biochemistry and Microbiology, University of Venda, Private Bag X5050, Thohoyandou 0950, South Africa; 3 Department of Biochemistry, Stellenbosch University, Stellenbosch 7600, South Africa; 4 Department of Anatomical Pathology, School of Pathology, University of the Witwatersrand/National Health Laboratory Service, Johannesburg 2000, South Africa

**Keywords:** breast cancer, cholesterol, cholesterol-depletion, cyclodextrins: 2-hydroxypropyl-β-cyclodextrin (HPβCD), anticancer agents

## Abstract

**Simple Summary:**

Breast cancer is a global burden with the most severe subtype being triple negative breast cancer (TNBC). Despite advances in conventional therapies, treatment for TNBC is currently lacking. Interestingly, cholesterol has gained interest as a potential therapeutic target due to cancer cells’ increased reliance on this macromolecule. In this study we aimed to assess the effects of cholesterol depletion as a therapeutic target in TNBC. We show that treatment with the cholesterol-depletory agent 2-hydroxypropyl-β-cyclodextrin (HPβCD) impeded the growth of cancer cells and further led to cancer cell death, which could be attributed to an altered cellular cholesterol profile following treatment. Furthermore, mice xenograft studies indicated complete eradication of early-stage tumours with no relapse, followed by a remarkable reduction in intermediate- and late-stage tumours, respectively. We have also identified SFRP1 as a possible molecular target facilitating the therapeutic action of HPβCD. These findings consequently potentiate cholesterol depletion as a novel anticancer strategy to be pursued.

**Abstract:**

Cholesterol accumulation is documented in various malignancies including breast cancer. Consequently, depleting cholesterol in cancer cells can serve as a viable treatment strategy. We identified the potency of 2-hydroxypropyl-β-cyclodextrin (HPβCD), a cholesterol-depletor in vitro against two breast cancer cell lines: MCF-7 (Oestrogen-receptor positive, ER+) and MDA-MB-231 (Triple negative breast cancer (TNBC)). The results were then compared against two non-cancerous cell lines using cytotoxic-, apoptosis-, and cholesterol-based assays. Treatment with HPβCD showed preferential and significant cytotoxic potential in cancer cells, inducing apoptosis in both cancer cell lines (*p* < 0.001). This was mediated due to significant depletion of cholesterol (*p* < 0.001). We further tested HPβCD in a MF-1 mice (*n* = 14) xenograft model and obtained 73.9%, 94% and 100% reduction in tumour size for late-, intermediate-, and early-stage TNBC, respectively. We also detected molecular-level perturbations in the expression patterns of several genes linked to breast cancer and cholesterol signalling pathways using RT^2^-PCR arrays and have identified SFRP1 as a direct binding partner to HPβCD through SPR drug interaction analysis. This work unravels mechanistic insights into HPβCD-induced cholesterol depletion, which leads to intrinsic apoptosis induction. Results from this study potentiate employing cholesterol depletion as a promising unconventional anticancer therapeutic strategy, which warrants future clinical investigations.

## 1. Introduction

Breast cancer (BC) is the most diagnosed cancer globally and is the second leading cause of cancer death in women [1]. Approximately 70–75% of invasive BC’s are characterized by the presence of the hormone receptors (ER^+^, PR^+^ and HER2), thus making them amenable to targeted chemotherapies. On the other hand, Triple negative breast cancer (TNBC) is characterized by the absence of the oestrogen receptor (ER), progesterone receptor (PR), and HER2 receptor, further constituting 20% of all BC cases [2]. Interestingly, TNBC most commonly occurs in females of African descent [3]. Oestrogen drives the development of ER^+^ breast tumours with its capacity to promote both proliferation and BC cells’ survival. Contrarily, the TNBC subtype is able to achieve malignancy in a hormone-independent manner, implicating several genes involved in cell-cycle regulation, DNA-damage response as well as metabolic signalling in disease pathogenesis [4]. The potential for metastasis in TNBC is quite similar to that of other BC’s, but these tumours display shorter median times for tumour relapse and patient survival due to their highly aggressive nature [5]. If cancer is detected at an early stage, the TNBC subtype is typically treated with chemotherapy drugs, radiation, and surgery, which unfortunately display severe side effects [6]. Most importantly, resistance to chemotherapeutic drugs hinders the treatment of cancer patients [7], with only 50% of the patient population manifesting a complete or partial response to therapy [8]. Consequently, alternative therapeutic approaches need to be investigated for successful cancer treatments.

One such approach is targeting cellular cholesterol. Cancer cells in general, require elevated levels of cholesterol to support cell proliferation. Additionally, cholesterol plays a crucial role in the maintenance of membrane rigidity as well as the formation of lipid rafts, which serve as key cancer signalling hubs [9]. Cellular cholesterol levels are largely regulated by biosynthesis, import, metabolism and export [10]. Synthesis is achieved in a highly regulated manner, following 21 enzymatic reactions in the mevalonate pathway [11]. Initially, mitochondrial-derived citrate is converted to acetyl-coenzyme-A, which is subsequently metabolized to lanosterol in a series of reactions in the endoplasmic reticulum [12]. The enzyme 3-hydroxy-3-methyl-glutaryl coenzyme A reductase (HMGCR) is crucial as it catalyses the rate-limiting step of cholesterol biosynthesis. Cholesterol is ultimately formed when lanosterol is converted to cholesterol either by the Bloch or Kandutsch–Russel pathway [13]. Importantly, upon carcinogenesis, the finely tuned mechanisms of cholesterol regulation are altered based on the energetic requirements of the cancerous cells [7]. Experimental discoveries propose a clear need for higher levels of cholesterol accumulation, which is facilitated through increased levels of cholesterol biosynthesis and uptake in malignant cells [14]. Additionally, reduced cholesterol efflux and increased influx leads to intracellular cholesterol dyshomeostasis [15], which plays a role in cancer progression and metastasis [7]. For instance, breast and prostate cancer cell lines have been shown to maintain higher levels of cholesterol and were seen to be sensitive to cholesterol-depletion-mediated cell death [16]. Furthermore, cholesterol is a known precursor of vitamin D and oestrogen, both of which play a key role in the histology of BC [15]. Consequently, focusing on the synthesis, transport, or metabolites of the cholesterol homeostasis pathways are possibilities for controlling malignant growth and development [17].

Currently, there are two therapeutic strategies that may be employed to target cholesterol for developing successful therapies in the treatment of BC. The first being cholesterol synthesis inhibitory drugs that involves the stunting of cholesterol synthesis by primarily targeting the rate-limiting enzyme HMGCR. This has been predominantly achieved by employing the most widely used lipid-lowering agent on the market, i.e., statins [18]. The second strategy is to decrease membrane cholesterol content by employing cholesterol-depleting agents. Over the last couple of decades several agents have been found to possess cholesterol-depleting properties [17]. The particular class of interest in this study is cyclodextrins (CyDs). The external environment of CyDs is hydrophilic, whereas the internal is hydrophobic, which is utilized to encapsulate lipophilic compounds such as cholesterol [19]. CyDs have also been shown to act as excipients due to their ability to improve drug solubility in aqueous solutions [20]. They are currently being marketed as a component of the formulations of over 35 marketed drugs. The most widely recognized CyDs utilized are: α-, β-, and γ-cyclodextrin. CyDs have also been shown to associate with cholesterol and phospholipids, thereby causing haemolysis [21]. A variant of CyDs, known as methyl-β-cyclodextrin (MBβCD) has been significantly utilized as a disrupting agent for lipid rafts through the extraction of cholesterol [22]. Several in vitro studies have exhibited that MβCD induces apoptosis of different cancer cell lines [23,24,25,26]. In contrast to MβCD, 2-hydroxylpropyl-β-cyclodextrin (HPβCD) displays enhanced solubility properties [27] and is well tolerated when dosed orally or intravenously, displaying minimal side effects with minor biochemical and histopathological alterations. A study documented that treatment with HPβCD led to reduced cholesterol intracellularly, resulting in a reduction in leukemic cell development through G_2/M_ cell-cycle mitotic arrest and apoptosis [26]. Furthermore, HPβCD prevented EMT from inhibiting the TGFβ/Smad signalling pathway, which consequently activates endoplasmic reticulum stress in the MDA-MB-231 cell line. Following the administration of HPβCD, TβR1 expression was significantly decreased, leading authors to postulate that treating cells with HPβCD affected the distribution of TβR1 in lipid rafts [28]. Additionally, HPβCD displayed minimal toxicity, contingent upon the dosage in several species [29]. HPβCD has additionally shown to be far less cytotoxic when contrasted with MβCD, while effectively removing cellular cholesterol [30]. A newly concluded clinical trial administering HPβCD for Niemann–Pick disease type C1 (NPC) (autosomal recessive lysosomal storage disorder due to the accumulation of unesterified cholesterol), yielded promising results. Currently, the drug is being tested in a multicentre, multinational Phase 2b/3 clinical efficacy trial [31]. Furthermore, HPβCD has been employed for compassionate use in NPC disease [32]. Therefore, we hypothesized and investigated HPβCD as a stand-alone compound without any complexation, potentiating its use as a promising therapeutic agent for BC.

## 2. Materials and Methods

**Figure 1:**
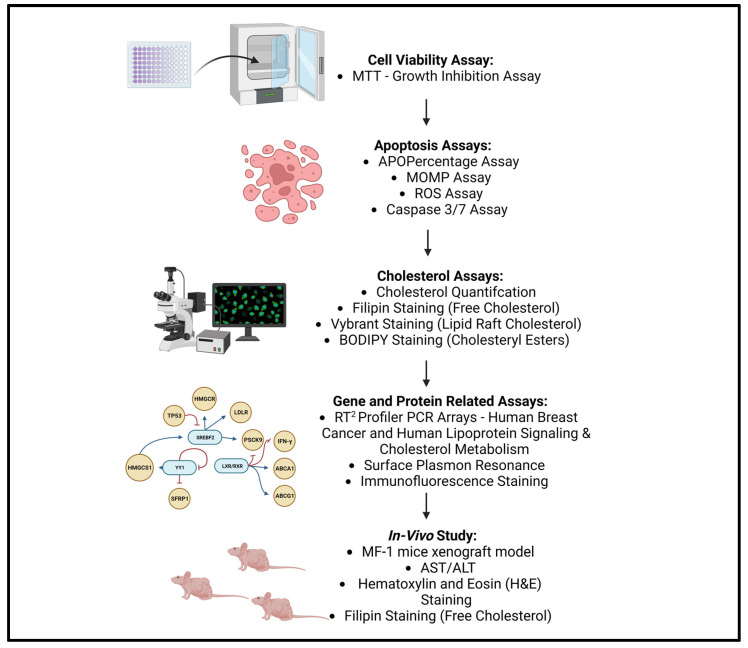
Schematic Summary of Study Workflow (Created using Bioreder.com (accessed on 7 May 2023)).

The methodology used in this study is summarised in Figure 1. Initially cell viability and apoptosis induction were measured in cancerous cell lines followed by the cellular cholesterol profiling post-cholesterol depletion, which was compared with healthy control non-cancerous cell lines to understand the role cholesterol plays in supporting BC survival and malignancy. This was subsequently followed by a mechanistic investigation into gene expression alterations in BC cells following cholesterol depletion along with a drug–protein interaction analysis of selected proteins. Finally, an in vivo MF-1 mice xenograft study was completed to validate in vitro findings, to measure the effect of HPβCD on tumour growth reduction and to monitor toxicity. Experiments were performed in triplicates, employing both biological and technical replicates, unless otherwise stated, to validate reproducibility and to facilitate statistical analyses.

### 2.1. Cell Culture

Four cell lines were used in the present study: tumorigenic cell lines, including hormone-positive breast cancer (MCF-7, The European Collection of Authenticated Cell Cultures (ECACC)) and TNBC cells (MDA-MB-231, Fox Chase Cancer Center); and non-tumorigenic or control cell lines, including human lung fibroblasts (MRC 5, Fox Chase Cancer Center) and human embryonic kidney cells (HEK-293, American Type Culture Collection (ATCC)) (either non-cancerous or control). MCF-7, MRC-5 and HEK-293 cells were cultured in Dulbecco’s Modified Eagle Medium (DMEM) (Thermo Fisher Scientific, Waltham, MA, USA). MDA-MB-231 cells were cultured in 3 parts DMEM to 1 part Ham’s F12 Nutrient Mixture (Thermo Fisher Scientific, Waltham, MA, USA). All media was supplemented with 10% FBS (Biowest, Nuaillé, France) and 1% penicillin-streptomycin (Thermo Fisher Scientific, Waltham, MA, USA). All four cell lines were cultured at 5% CO_2_ in a 37 °C incubator (Thermo Fisher Scientific, Waltham, MA, USA). Mycoplasma monitoring was conducted using the MycoFluor™ Mycoplasma Detection Kit (Thermo Fisher Scientific, Waltham, MA, USA).

### 2.2. Preparation of Drug Treatments

10 mM stock solutions of MβCD (Sigma-Aldrich, Dorset, UK) and 100 mM stock solutions of HPβCD (Sigma-Aldrich, Dorset, UK) were prepared by dissolution in purified water. These were further diluted in media to achieve a treatment dose of 5 mM and 10 mM, respectively. Additionally, 50 mM stock solution of Plumbagin (PL) was dissolved in DMSO (Sigma-Aldrich, Dorset, UK) and further diluted in media to achieve the treatment dose of 40 μM. PL was utilized as a positive control based on previous studies indicating its cytotoxic effects [33]. Dosage and time points were determined from preliminary experiments.

### 2.3. Growth Inhibition

Growth inhibition was estimated using the MTT assay (Sigma-Aldrich, Dorset, UK) as previously described [34]. A range of concentrations of HPβCD (Sigma-Aldrich, Dorset, UK) was tested in tumorigenic and control non-tumorigenic cell lines (5000 cells/well in 96-well plate) as indicated in the results for either 24, 48, or 72 h. The appropriate concentration of 10 mM for downstream assays was selected based on the results of the MTT assay. Control wells had equivalent volumes of 40 μM PL (Sigma-Aldrich, Dorset, UK). The optical density (OD) was subsequently measured at 570 nm using a Multiskan GO Microplate Reader using the SkanIt™ Software 2.0 (Thermo Fisher Scientific, SkanIt™ software).

### 2.4. Cell Death Experiments

Cells (5000/well in 96-well plate) were treated with various concentrations of HPβCD and the respective controls, 5 mM MβCD and 40 μM PL, as a positive control for 24 h. Media was removed and cells were stained with an APOPercentage™ (Biocolor, Carrickfergus, UK) dye as previously described [35]. OD was measured at 550 nm.

Mitochondrial outer membrane potential (MOMP) assay was performed as described previously [35]. Cells were treated for 24 h with a concentration of 10 mM HPβCD, and 10 mM H_2_O_2_ was used as a positive control. Staining was done with a JC-1 dye (5,5′,6,6′-tetrachloro-1,1′,3,3′-tetraethylbenzimidazolylcarbocyanineiodide) (Sigma Aldrich, UK) for 30 min. Analysis using the BD Accuri™ C6 flow cytometer and software (BD Biosciences, Piscataway, NJ, USA) was performed by plotting FL2-A vs. FL1-A and applying a quadrant gate to determine JC-1 aggregates and monomers.

Reactive oxygen species (ROS) assay was performed as described [36]. Cells were treated for 24 h with a concentration of 10 mM HPβCD. 10 mM H_2_O_2_ was used as a positive control. Staining was done with 2′,7′–dichlorofluorescin diacetate (DCFDA) dye for 1 h. Analysis using the BD Accuri™ C6 flow cytometer (Software Version 264.21) (BD Biosciences, Piscataway, NJ, USA) was performed by plotting a 1D plot of the FL1 channel.

Caspase-3/7 assay was performed using Muse^®^ Caspase-3/7 Assay Kit (Merck, Branchburg, NJ, USA) as per the manufacturer’s protocol. 10 mM HPβCD was administered for 24 h. 10 μM PL was used as a positive control for the MCF-7 cell line only (because at higher concentrations, apoptosis could not be captured). 40 μM PL was used as a positive control for the remaining cell lines. The samples were subsequently analysed using the Muse™ Cell Analyser (Merck, Branchburg, NJ, USA) with appropriate gates to eliminate cell debris.

### 2.5. Cholesterol Staining

Cells were treated with 10 mM HPβCD for 24 h. Positive control wells had equivalent volumes of 5 mM MβCD (Sigma-Aldrich, UK). Following treatment, cholesterol was stained with several fluorescent dyes and quantified. Namely, Filipin (Sigma-Aldrich, UK) for free cholesterol, CholEsteryl BODIPY™ FL C12 (Thermo Fisher Scientific, USA) for cholesteryl esters (CEs), and the Vybrant™ Alexa Fluor™ 594 Lipid Raft Labelling Kit (Invitrogen™, Thermo Fisher Scientific, UK) for lipid rafts. Nuclei were stained with either DAPI (Sigma-Aldrich, UK) or NucRed^®^ Dead 647 ReadyProbes^®^ Reagent (Thermo Fisher Scientific, USA). Mice tumours from three different groups were sectioned, dewaxed, and stained with Filipin as mentioned in results. Cell/tumour sections were visualised using the FLoid™ Cell Imaging Station (Thermo Fisher Scientific, Swindon, UK) and densitometry analysis was performed using the ImageJ Software (Software Version 1.41) (NIH, Bethesda, MD, USA).

### 2.6. RT^2^ Profiler™ PCR Arrays

Human Breast Cancer and Human Lipoprotein Signaling & Cholesterol Metabolism RT^2^ Profiler™ PCR arrays (Qiagen, Hilden, Germany) were used post treatment. RNA was extracted, and cDNA synthesis was performed using the RT^2^ First Strand Kit (Qiagen, Hilden, Germany) as per the manufacturer’s protocol. Synthesized cDNA samples were mixed with the RT^2^ SYBR Green qPCR Master mix (Qiagen, Hilden, Germany) and added to the appropriate array of interest, after which RT-qPCR was performed with appropriate controls using the CFX96 Touch™ Real-Time PCR Detection System (Bio-Rad, Hercules, CA, USA). Analysis was performed using the RT^2^ Profiler™ PCR Array Data Analysis v3.5 software (Qiagen, Hilden, Germany). Relative changes in gene expression were analysed using the 2^–ΔΔCT^ method, comparing the treated to the untreated group. Differentially expressed genes were identified as those with a |log_2_ (fold-regulation)| ≥1 against the untreated group. Heatmaps were generated using MultiExperiment Viewer (MeV 4.9.0) (J. Craig Venter Institute, La Jolla, CA, USA) [37] to visualize differentially expressed genes with log_2_ fold change. OmicsNet (Institute of Parasitology, Quebec, QC, Canada) was used to identify protein–protein interactions of the differentially expressed genes [38], and networks of molecular interactions and biological pathways were created using Cytoscape (Software Version 3.7.2) (Institute for Systems Biology, Seattle, WA, USA) [39].

### 2.7. Determination of Binding Affinity of Selected Proteins for HPβCD

The binding affinity of HPβCD with six human proteins (chosen based on significant fold change in RT^2^ Profiler™ PCR Arrays and literature-based search defining their importance in cancer) was conducted using the BioNavis™ 420A ILVES MP-SPR (BioNavis, Tampere, Finland) at 25 °C. As running buffer, degassed PBS Tween 20 was used. The recombinant proteins were immobilized as ligands at 0.5 ug/mL onto functionalized 3D carboxymethyl dextran sensors (CMD 3D 500 L; BioNavis, Tampere, Finland). The immobilization of ligands was achieved through amine coupling after 1-Ethyl-3-(3-dimethylaminopropyl) carbodiimide (EDC) (Sigma Aldrich, Germany) and N-Hydroxy-succinimide (NHS) (Sigma Aldrich, Germany) activation following a protocol provided by the manufacturer (BioNavis, Finland) to achieve <200 Rus. A reference channel without immobilized protein served as a control for non-specific binding and changes in refractive index. As for an analyte, HPβCD was prepared into aliquots of 0, 1.25, 2.5, 5 and 10 nM injected three times at a flow rate of 50 μL/min into each flow cell. Injections with buffer only were used as controls. Association between ligand and analyte was allowed for 3 min, and dissociation was monitored for a total of 10 min. Kinetics steady-state equilibrium constant data was processed after the double referencing of the sensor grams and concatenating the responses of all five analyte concentrations by global fitting using TraceDrawer software version 1.8 (Ridgeview Instruments, Uppsala, Sweden).

### 2.8. Immunofluorescent Staining of MDA-MB-231 Cells

MDA-MB-231 cells were treated with 10 mM HPβCD for 24 h at 37 °C. Following treatment, the media was removed, and cells were washed with PBS (Thermo Fisher Scientific, USA). Cells were subsequently fixed using 4% paraformaldehyde (Sigma Aldrich, UK) at room temperature for 10 min. Cells were then permeabilized by incubating in 0.1% Triton-X (Sigma Aldrich, UK) for 15 min. Thereafter a one-hour blocking step was completed by incubating cells with 1% bovine serum albumin (BSA) (VWR Life Sciences, Radnor, PA, USA) made up in 0.1% PBS-Tween 20 (PBST). Following this, primary antibody was added (Rabbit anti-human SFRP1 (Abcam Boston, MA, USA); (ab4193—1:250 dilution)) and incubated at 4 °C overnight. Once the incubation was complete, the secondary antibody was subsequently added (Goat Anti-Rabbit Texas Red (Thermo Fisher Scientific, USA); (T-2767—1:1000 dilution)) and incubated for a duration of 45 min. For nuclear staining, 0.0001 mg/mL DAPI (Sigma Aldrich, UK) was administered. Following the addition of ProLong™ Gold Antifade mounting media (Thermo Fisher Scientific, USA) to the slides, cells were subsequently visualized by using the Floid™ Cell Imaging System (Thermo Fisher Scientific, USA). Immunofluorescence intensity was quantified by utilizing the ImageJ software (Software Version 1.41) (NIH, Bethesda, MD, USA). 

### 2.9. In Vivo Xenograft Study

A non-blinded study was performed. 14 MF-1 (age: 5–8 weeks old, *n* = 14) female nude mice were used for the study and the animals were habituated for one week at the Wits Research Animal Facility, Parktown Campus, Johannesburg, South Africa. The main objective was testing the effects of HPβCD on tumour growth and its side effects. The study was subdivided into 4 groups: *(1)* Mice injected with MDA-MB-231 cells (*n* = 4) but not treated with HPβCD to serve as control. These mice were injected with PBS only (Untreated, average tumour size ~13,000 mm^3^). *(2)* Mice injected with MDA-MB-231 cells (*n* = 4) and treated with HPβCD (Late stage, tumour size ~3500 mm^3^) (*n* = 4). *(3)* Mice injected with MDA-MB-231 cells (*n* = 3) and treated with HPβCD (Intermediate-stage, tumour size ~800 mm^3^) (*n* = 3). *(4)* Mice injected with MDA-MB-231 cells (*n* = 3) and treated with HPβCD (Early stage, tumour size ~20 mm^3^) (*n* = 3). 3 million MDA-MB-231 cancer cells prepared in sterilized PBS (100 μL) were injected subcutaneously into the left flank of the MF-1 nude mice, thereby resulting in tumour growth. Tumour xenografts were measured using callipers in two dimensions (2D) thrice a week until the tumours reached a certain size. A maximum of 3000 mg/kg body weight of HPβCD was injected intraperitoneally thrice a week over a period of over 3 weeks for a maximum number of 10 doses. The response to therapy was evaluated by investigating the effects of HPβCD on the weight of the mouse, growth rate, and shrinkage/regression of the tumour. Following treatment duration, mice were euthanised using Eutha-naze (Bayer (Pty) Ltd. Animal Health Div., South Africa) containing Sodium Pentobarbitone. At termination, mice were necropsied; blood was collected for alanine aminotransferase (ALT) and aspartate aminotransferase (AST) measurements. Tumour size was expressed in mm^3^ using the formula V = 1/2 × a × b^2^, where ‘a’ and ‘b’ represent the long and short diameters of the tumour, respectively. The tumour mass was weighed and photographed at the end of the experiment after being harvested. The tumour mass was cut into small pieces and snap frozen in liquid nitrogen for RNA extraction. The tumour inhibition rate was calculated as Inhibition rate (%) = (average tumour volume of control group-average tumour volume of test group)/(average tumour volume of control group) × 100%. Blood was collected post-euthanisation by perfusion of mice, and serum was separated. The serum was then tested for AST/ALT liver toxicity using IDEXX Catalyst Dx Chemistry Analyzer (IDEXX, Westbrook, ME, USA) with individual AST/ALT cartridges.

### 2.10. Hematoxylin and Eosin (H&E) Staining of Mice Tumours

Tissue sections were cut and stained with H&E (Department of Anatomical Pathology, National Health Laboratory Service, Johannesburg, South Africa) as per operating procedure. The analysis was performed by a pathologist.

### 2.11. Statistical Analyses

Statistical analysis was performed using Microsoft Office Excel© and GraphPad Prism version 8 (San Diego, CA, USA). The statistical significance of differences between control and treated sample cells were calculated using the Student’s *t*-test and one-way analysis of variance (ANOVA) followed by a Bonferroni post hoc test. A *p*-value of <0.05 (*) was the critical value for significance. A Z-factor was also calculated for each assay (above >0.6) performed in 96-well plates, indicating good to excellent robustness [40].

## 3. Results

### 3.1. HPβCD Reduces Cellular Growth and Proliferation Via Apoptosis

**Figure 2:**
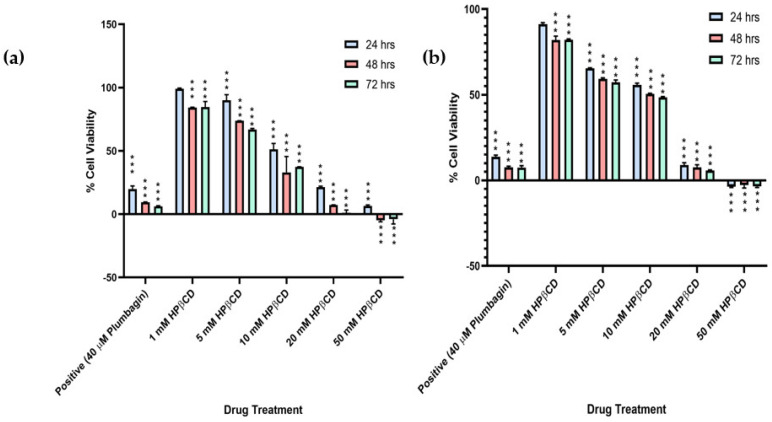

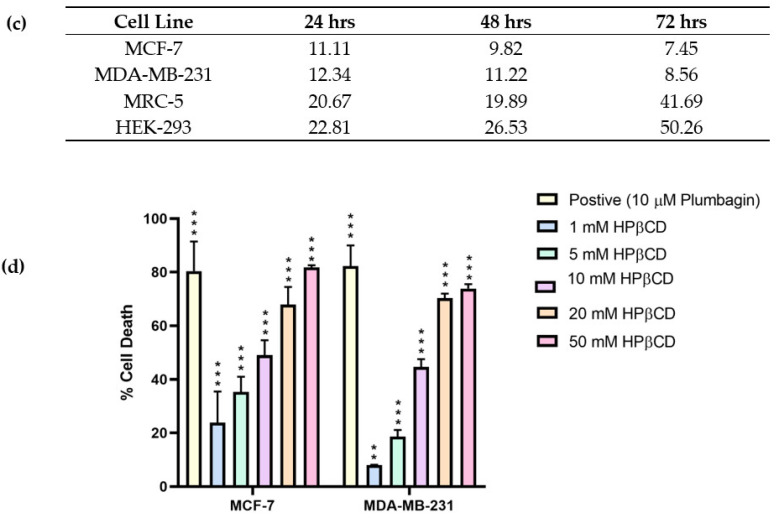
Graph comparing the percentage growth inhibition of (**a**) MCF-7 and (**b**) MDA-MB-231 cells at selected concentrations of HPβCD at 1 mM, 5 mM, 10 mM, 20 mM, and 50 mM relative to the untreated control. PL (40 μM) was used as a positive control. (**c**) IC_50_ (mM) values for MCF-7, MDA-MB-231, MRC-5, and HEK-293 cells in mM. (**d**) Graph comparing the percentage of apoptosis in MCF-7 and MDA-MB-231 cells at selected concentrations of HPβCD at 1 mM, 5 mM, 10 mM, 20 mM, 50 mM. PL (10 μM) was used as a positive control. A one-way ANOVA test was performed for statistical analysis. A Bonferroni post hoc test was employed for pairwise analysis comparing treated groups to the untreated groups. Data represents mean ± standard deviation S.D. (*n* = 3) from raw data, where ** *p* < 0.01, and *** *p* < 0.001 indicates significant difference to untreated control. Independent experiments were run at least three times.

To investigate the potential of HPβCD in cellular growth inhibition, an MTT assay was performed. Results obtained for MCF-7 and MDA-MB-231 cells showed ~50% growth inhibition at 10 mM concentration of HPβCD (Figure 2a,b). Subsequently, the concentrations of HPβCD administered dose-dependently inhibited growth of both cell lines. The result was consistent for all the three time points (24 h, 48 h, 72 h), thus indicating its potential ability to disrupt the mitochondrial processes and its probable slow action (Figure 2). This is in accordance with previous papers published for leukaemia [26] and atherosclerosis [41]. On the contrary, the non-cancerous control cell lines MRC-5 and HEK-293, showed no growth inhibition at the highest dose administered (50 mM HPβCD) for both 24 h and 48 h, respectively (Figure S1). Upon calculating the IC_50_ values, it was seen that almost double the concentration of HPβCD was required to induce 50% growth inhibition in both the cell lines at 24 h (Figure 2c), and at 72 h, the concentration increased to almost five times as compared to the cancerous cells, thus indicating that HPβCD might be specifically toxic to cancerous cells.

Based on the MTT assay, a single time point of 24 h was selected for further downstream assays to investigate if cell growth inhibition was due to cell death. An APOPercentage assay was performed to confirm cell death via apoptosis. Results for MCF-7 and MDA-MB-231 cells showed about 50% cell death at 10 mM concentration of HPβCD. The cell death percentage was proportional to an increase in concentration of HPβCD across all the treatments (Figure 2d). Non-cancerous cell lines showed negligible cell death at the highest dose administered (10 mM HPβCD) (Figures S2 and S3). Even at higher concentrations of 20 mM and 50 mM, the maximum apoptosis observed in control cells was ~20% for both the cell lines. This consequently suggests that HPβCD is less toxic to non-cancerous cells.

**Figure 3:**
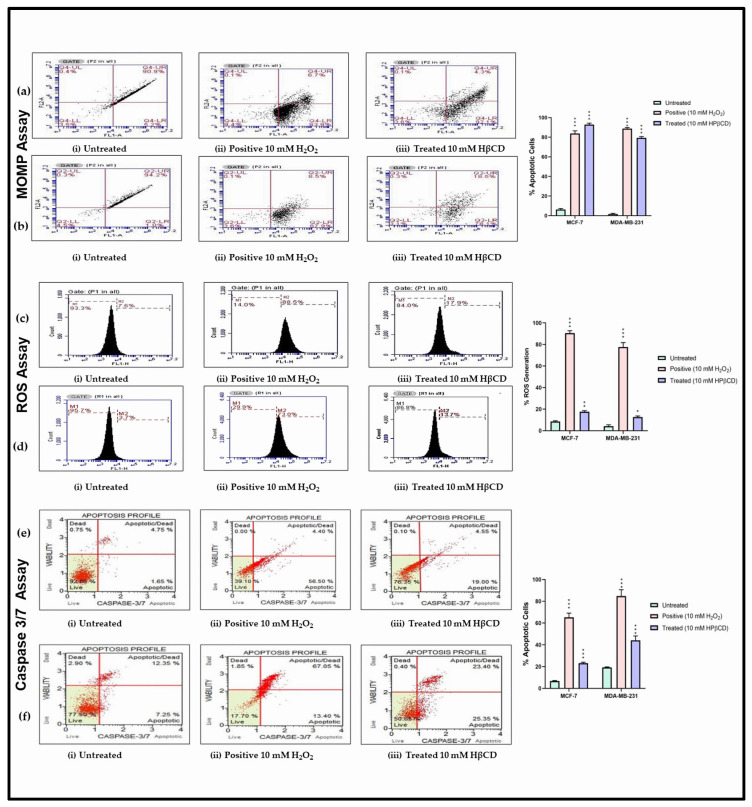
MOMP plots for (**a**) MCF-7 and (**b**) MDA-MB-231 cells; (i) loss of mitochondrial membrane potential in untreated cells, (ii) in positive sample (10 mM H_2_O_2_), and (iii) at 10 mM HPβCD. (**c**) ROS generation in MCF-7 cells; (i) in untreated cells (7.6%), (ii) in positive sample (10 mM H_2_O_2_) (88.5%), and (iii) at 10 mM HPβCD (17.9%). (**d**) ROS generation in MDA-MB-231 cells; (i) in untreated cells (3.7%), (ii) in positive sample (10 mM H_2_O_2_) (73%) and (iii) at 10 mM HPβCD (13.7%); (**e**) Caspase-3/7 activity in MCF-7 cells; (i) total apoptotic/live cells generation in untreated cells, total apoptotic—6.4%, (ii) total apoptotic/live cells generation in positive sample (10 μM PL), total apoptotic—60.90%, and (iii) total apoptotic/live cells in treated (10 mM HPβCD) cells, total apoptotic—23.55%. (**f**) Caspase-3/7 activity in MDA-MB-231 cells; (i) total apoptotic/live cells generation in untreated cells, total apoptotic—19.6%, (ii) total apoptotic/live cells generation in positive sample (40 μM PL), total apoptotic—80.45%, and (iii) total apoptotic/live cells generation in treated (10 mM HPβCD) cells, total apoptotic—48.75%. A one-way ANOVA test was completed for statistical analysis. A Bonferroni post hoc test was employed for pairwise analysis, comparing treated groups to the untreated groups. Data represents mean ± standard deviation (SD) (*n* = 3) from raw data, where * *p* < 0.05, ** *p* < 0.01, and *** *p* < 0.001 indicates significant difference to untreated control.

One of the key hallmarks of apoptosis is the loss of MOMP [42]. To investigate whether MOMP is the basis for the observed apoptosis in cancer cells, a MOMP assay was performed using JC-1 dye. From the results obtained from MTT (IC_50_ values) and APOPercentage assays, it was evident that ~10 mM HPβCD at 24 h caused cellular growth inhibition through apoptosis in cancer cells and had a minimal effect in non-cancerous cells (*p* < 0.001). Therefore, 10 mM HPβCD treated for 24 h was selected going forward as the only working concentration and time point for HPβCD treatment. Results obtained for MCF-7 and MDA-MB-231 (Figure 3a,b) showed 92.1% and 78.1% apoptotic population, respectively at 10 mM concentration of HPβCD. For the control MRC-5 cell line, 98.2% of the cell population was non-apoptotic/healthy in the untreated sample (Figure S4a), and 12.8% apoptotic cells were evident when treated with 10 mM concentration of HPβCD (Figure S4a). This confirms that HPβCD is much less toxic to non-cancerous cells, while being significantly apoptotic to cancerous cells via the intrinsic apoptotic pathway and causes loss of MOMP. This is possibly due to the nature of CyDs in general as cholesterol chelators and their ability to deplete mitochondrial cholesterol content as demonstrated for MβCD [43]. Moreover, cancer cells in general have enhanced metabolism since they divide rapidly as compared to normal cells, thereby leading to an abundant generation of ROS [44]. Therefore, to further validate the induction of apoptosis and to investigate the exact mechanisms of apoptosis in this study, a ROS assay was performed. The ROS production in MCF-7 and MDA-MB-231 cells following 10 mM HPβCD treatment was 17.9% and 13.7%, respectively (Figure 3c,d). Even though this was about 2.5- to 4-fold more compared to the untreated sample, the effect was not too drastic considering that the levels of ROS generation in control MRC-5 cells showed 10.8% ROS activity at 10 mM HPβCD treatment (Figure S4b). Thus, it can be inferred that although HPβCD does cause apoptosis, it does not do so via an enormous production of ROS. Downregulation of ROS in apoptosis has often been observed considering that ROS has significant roles in physiological processes [45]. It is quite possible that there is an over expression of detoxifying enzymes, such as catalase and glutathione reductase, which are responsible for the maintenance of free radicals. As a final step to confirm and validate HPβCD’s mechanism of apoptosis via the intrinsic pathway, the caspase-3/7 assay was performed. This was completed on the basis that CyDs have been previously implicated in caspase activation [46]. For MCF-7 and MDA-MB-231 cell lines, the total apoptosis observed in the treated cells (10 mM HPβCD) increased by 23.55% and 48.75%, respectively (Figure 3e,f), which was almost four times more compared to the untreated sample. The overall low caspase-3/7 activity in MCF-7 cells was due to the fact that MCF-7′s do not express caspase-3 activity in general [47]. This assay gives a measure of both caspase-3 and caspase-7; therefore, the cell death activity in MCF-7 was solely due to caspase-7 and, quite possibly, caspase-7 activity in MCF-7 cells was low overall. In the control HEK-293 cells, the total healthy population in the untreated cells was 71.90% (Figure S4c), which decreased only partially in the 10 mM HPβCD treated sample (Figure S4c) and decreased to 66.75%, which further warrants the fact that HPβCD is non-toxic to non-cancerous cells.

### 3.2. HPβCD Induces Apoptosis in Cancer Cells via Cholesterol Depletion

**Figure 4:**
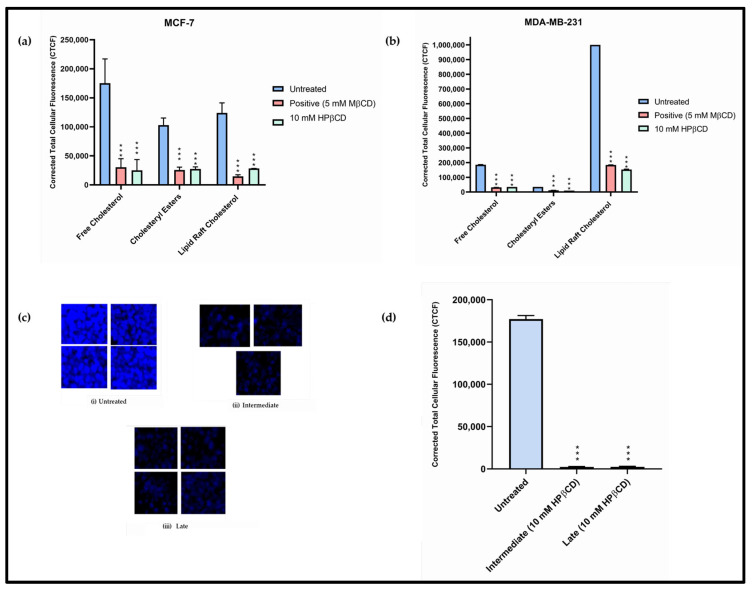
Filipin staining of (**a**) MCF-7 and (**b**) MDA-MB-231 cells to determine free cholesterol content. Lipid droplet staining of (**a**) MCF-7 and (**b**) MDA-MB-231 cells to determine cholesteryl ester content. Lipid raft staining of (**a**) MCF-7 and (**b**) MDA-MB-231 cells to determine lipid raft cholesterol content. Comparison of staining in untreated, positive (5 mM MβCD) and treated (10 mM HPβCD). The scale bar on each of the images represents 100 μM. (**c**) Filipin staining (for cholesterol) of mice sections: untreated (*n* = 4), intermediate- (*n* = 3) and late-stage (*n* = 4). (**d**) Results show decreased cholesterol in intermediate and late-stage tumour compared to untreated. A one-way ANOVA test was completed for statistical analysis. A Bonferroni post hoc test was employed for pairwise analysis, comparing treated groups to the untreated groups. Data represents mean ± standard deviation (SD) (*n* = 3) from raw data, where *** *p* < 0.001 indicates significant difference to untreated control.

Cholesterol is crucial to cancer cell survival and proliferation and forms an integral part of the cell membrane. On this basis, it was hypothesized that HPβCD, through its cholesterol depleting properties, extracts excess cholesterol from cancer cells and, in this way, induces apoptosis. To explore this, a preliminary test was completed employing a cholesterol assay, which provided an estimate of total cholesterol, CEs and free cholesterol and confirmed results as obtained for leukaemia [26] and atherosclerosis [41]. Results for the MCF-7 cell line (Figure S5) clearly showed that, compared to untreated, the HPβCD-treated cells had reduced total cholesterol content at all concentrations tested (1 mM, 5 mM, 10 mM, 20 mM, and 50 mM). At 10 mM, which was the concentration selected based on previous assays, the total cholesterol level was reduced to more than 75% as compared to the untreated sample. The MDA-MB-231 cell line had lower level of cholesterol compared to MCF-7 (Figure 4 and Figure S5), but the results followed a similar trend to the MCF-7 cells. MRC-5 cells, being non-cancerous, displayed lower levels of cholesterol compared to both MCF-7 and MDA-MB-231 cell lines because they do not divide aggressively and, therefore, require less cholesterol (Figure S5). In MRC-5 cells, the total cholesterol levels were reduced compared to the untreated, but this depletion was not enough to reduce cellular viability as observed from earlier assays. Upon quantifying the total cholesterol content, it was seen that MCF-7 and MDA-MB-231 cell lines possess approximately 5- and 2-times increased cholesterol, respectively, as compared to the non-cancerous MRC-5 cells (Figure S5). After this preliminary assay, to further confirm HPβCD-mediated cholesterol-depletion, cells were stained with Filipin (Figure 4a,b and Figure S6), CholEsteryl BODIPY™ FL C12 (Figure 4a,b and Figure S6) and Vybrant™ Alexa Fluor™ 594 Lipid Raft Labelling Kit (Figure 4a,b and Figure S6). Additionally, mice tumour sections were stained with Filipin alone for final confirmation (Figure 4c). Both MCF-7 and MDA-MB-231 cell lines showed more than 70–80% reduction in cholesterol (CTCF) (Figure 4a,b and Figure S6). While treatment with MβCD significantly reduced cellular cholesterol profile in HEK-293 cells, HPβCD did not have a drastic effect on the cellular cholesterol profile, hence validating results obtained through cell viability (Figure S7). Interestingly, the mice tumour sections for intermediate and late-stage TNBC showed more than 99% reduction in cholesterol (Figure 4d), thus confirming HPβCD’s mode of action.

### 3.3. HPβCD Reduces Tumour Size in Mice with Reduced Chances of Relapse

**Figure 5:**
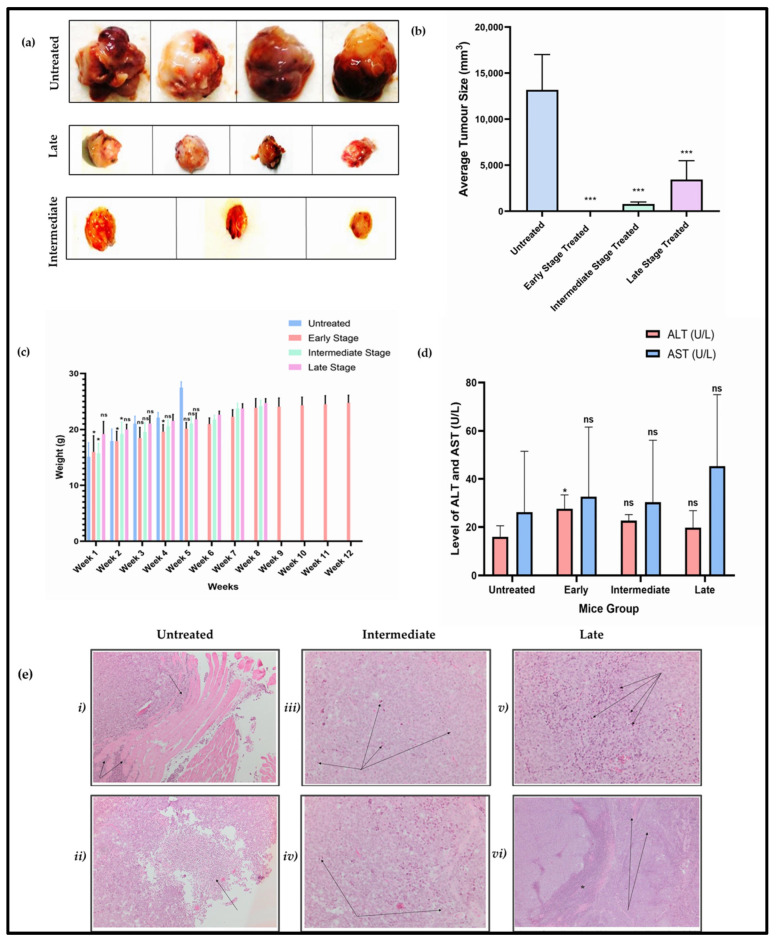
(**a**) Mice tumour images post-euthanisation showing tumour sizes in untreated group of the late, late-treated, and intermediate-stage tumours, injected with MDA-MB-231 cells and treated with HPβCD (3000 mg/kg bw) for 10 doses. (**b**) Results show tumour reduction by 73.9% compared to the untreated group in the late-stage group and a 94% reduction in the intermediate stage compared to the untreated group. (**c**) Weight change in all 14 mice across the study. Significance was only calculated for five weeks because the untreated group had to be euthanized at this point. (**d**) Graphical representation of AST/ALT levels in all 14 mice across the study. Permissible range in mice for ALT is 28-132 U/L and for AST is 59-247 U/L. (**e**) H&E examination of untreated, intermediate-, and late-stage in MDA-MB-231 mice. (i) Tumour cells beginning to dissect between skeletal muscle fibres (arrows). Original magnification 100×. (ii) An area of necrosis identified (arrows). Original magnification 100×. (iii) Numerous apoptotic cells (arrows). Original magnification 200×. (iv) A number of mitotic figures are present (arrows). Original magnification 200×. (v) Numerous apoptotic cells are identified (arrows). Original magnification 200×. (vi) A large area of geographic necrosis (asterisk) is present together with scattered necrotic foci (arrows). Original magnification 100×. A one-way ANOVA test was completed for statistical analysis. A Bonferroni post hoc test was employed for pairwise analysis, comparing treated groups to the untreated groups. Data represents mean ± standard deviation (SD) (*n* = 3 or *n* = 4) from raw data, where * *p* < 0.05 and *** *p* < 0.001 indicates significant difference to untreated control and ‘ns’ corresponds to insignificant difference to untreated control.

Since the efficacy of HPβCD against BC was established in in vitro assays, we further performed investigations in a mice model (MF-1 nude mice) as described in the methods section. Following approximately 4–5 weeks after cells were injected, tumours were observed in the untreated group (*n* = 4), and the average size increased to 13,178.12 mm^3^ in two weeks’ time. This is because MDA-MB-231 cells are extremely aggressive in nature [48]. These four mice had to be euthanized at this point because of humane end points. In the late-stage-treated group, the treatment commenced when the size of the tumour ranged between 900 mm^3^ to 1350 mm^3^ (Figure 5a,b). After administration of 10 doses of HPβCD, a significant reduction was observed in tumour growth and the average size of the tumour reduced to a significant 73.9% compared to the untreated group (Figure 5a,b). In the intermediate-stage group (*n* = 3), the treatment commenced when the size of the tumour reached ~250 mm^3^. Again, after 10 doses of the HPβCD treatment, the average size of the tumour decreased to 784.18 mm^3^, which is a 94% decrease (Figure 5a,b) as compared to the untreated group. This was remarkable considering the invasiveness of MDA-MB-231 cells. Finally, in the early-stage group (*n* = 3), the treatment commenced when the tumour size was ~20 mm^3^ (Figure S8a). After treatment with HPβCD, the tumours were completely healed (Figure S8b). In fact, the tumour shrank even before completing the 10 doses, but the treatment was continued to complete 10 doses regimen. These three mice were then kept for a period of 4 weeks to test for relapse of tumour because TNBC tumours have propensity to recur [6]. After 4 weeks of the relapse test (Figure S8c), no recurrence was observed. A significant reduction in/complete healing of tumours was also observed in four mice with MCF-7 xenograft early-stage tumours (Figure S12).

Additionally, no abnormal weight change was observed (Figure 5c) pointing to the optimal health of the mice under study. To further test for liver toxicity, mice serum was analysed for AST/ALT levels. (Figure 5d). The AST/ALT ratio for all the mice were under the recommended ratio of two and the values were within the reference ranges. The levels of AST/ALT in the late-, intermediate-, and early-stage mice were not significantly different as compared to the untreated group. Further, H&E staining for mice tumour sections indicated brisk mitotic activity in all three groups, with the untreated cases displaying numerous atypical mitotic figures, which were not observed in the intermediate- and late-stage groups. Additionally, numerous necrotic structures in late- and intermediate-stage tumours were observed compared to untreated (Figure 5e and Table S1). Microscopic examination revealed solid sheets of cohesive, markedly pleomorphic neoplastic cells. Untreated cases demonstrated extensive muscle infiltration. While the untreated cases only showed necrosis at the peripheries, the intermediate- and late-stage groups showed an increase in necrotic foci throughout the tissue sections. Collectively, the late-stage group showed more apoptosis. These findings suggest that treatment with HPβCD resulted in increased cell death in the intermediate- and late-stage groups in contrast to the untreated group. Therefore, in conclusion to the in vivo study, it can be potentially inferred that, possibly in humans, if the tumour is diagnosed at a late stage or intermediate stage, HPβCD treatment might slow the progression of the cancer and thereby improve human life expectancy and that if the cancer is detected at an early stage, the cancer might be completely curable without any severe side effects of the therapy.

### 3.4. HPβCD Differentially Regulates Genes in MCF-7 and MDA-MB-231 Cell Lines

Using the Human Breast Cancer (Figure S9a) and the Human Lipoprotein Signaling & Cholesterol Metabolism (Figure S9b) RT^2^ Profiler™ PCR Arrays, heatmaps representing the gene expression changes in multiple pathways involved in BC and cholesterol metabolism were observed after treatment with HPβCD in both cancerous cell lines. Interestingly, the gene expression profiles were opposite in these cell lines where MCF-7 showed downregulation of many genes, whereas upregulation was observed in MDA-MB-231 cells. This correlates with the aggressive phenotype of the MDA-MB-231. Figures S10 and S11 show the network of genes based on their involvement in various pathways and a large proportion of the genes were downregulated in treated sample when compared to the untreated sample. This suggests that HPβCD treatment has the capability to modulate several cancer-related and cholesterol metabolism/lipoprotein signalling pathways, thus contributing to understanding pathway dynamics underlying cholesterol-mediated cancer growth, progression, and drug response.

### 3.5. SFRP1: A Potential Drug-Binding Target for HPβCD?

**Figure 6:**
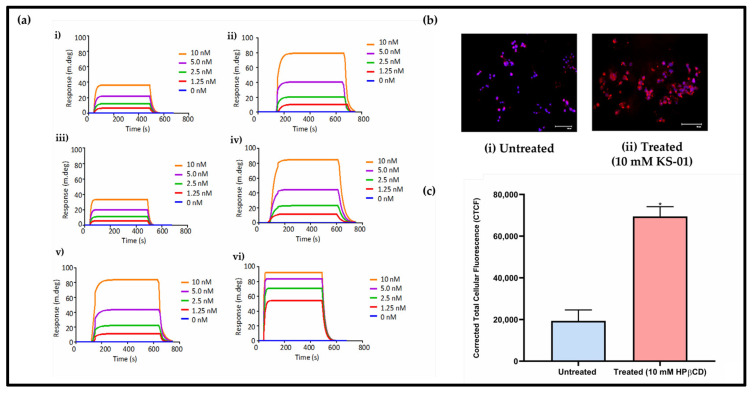
(**a**) HPβCD is capable of binding human proteins. Shown are the representative SPR sensor grams representing direct interaction of HPβCD with (i) ABCA1, (ii) ADAM23, (iii) AKT1, (iv) GATA3, (v) Cathepsin D, and (vi) SFRP1. The sensor grams were analysed to generate the kinetics in Table 1 (**b**) Protein expression of putative tumour suppressor SFRP1 in untreated and 10 mM HPβCD treated MDA-MB-231 cells for a duration of 24 h was detected by employing immunofluorescent microscopy. SFRP1 expression is indicated by Texas Red staining (red). (**c**) It is evident that SFRP1 is drastically increased following treatment with HPβCD. Images were captured using the Floid™ Cell Imaging System followed by analysis using the Image J software. The scale bar on each of the images represents 100 μM. A paired two-tailed *t*-test was employed for statistical analysis. Data are mean ± standard deviation (SD) (*n* = 3) from raw data, where * *p* < 0.05 corresponds to insignificant difference to untreated control.

Using a gene expression threshold of fold change more than +2 or less than −2 relative to untreated cells, cytoscape analysis (Figures S10 and S11), and these genes’ relevance for cancer and cholesterol-related processes, six genes were shortlisted, namely, ABCA1 (cholesterol efflux), ADAM23 (a possible tumour suppressor), AKT1 (a critical regulator of cell survival, proliferation, and metabolism), GATA3 (a possible oncogene or tumour suppressor), Cathepsin D (highly expressed in metastatic cancer), and SFRP1 (a tumour suppressor and a negative regulator of the Wnt pathway in cancer), which were then analysed for drug–protein interactions via SPR. The study was conducted to determine the steady-state HPβCD binding affinities with the BC marker recombinant protein versions. First, it was important to validate the Langmuir kinetic model used, the Chi-square values below 10 are suggestive of a good fit for 1:1 binding (Figure 6a). The SPR sensor grams generated (Figure 6a) were fit to a simple Langmuir kinetic binding model to calculate the association rate constant (*ka* in the unit of M^−1^s^−1^), dissociation rate constant (*kd* in the unit of s-1), and the equilibrium constant (*K_D_* in the unit of M) (Figure 6a and Table 1). Generally, it was observed that all the recombinant protein versions of the selected genes interacted with HPβCD in a concentration-dependent manner (Figure 6a). This suggests that the interactions were specific. Interestingly, SFRP1 had the highest *k_a_* and the lowest *K_D_* value (within nanomolar range) when compared to the other proteins (Figure 6a; Table 1). The binding affinities in our study were observed to be in the following order: SFRP1 > ABCA1 > AKT1 > GATA3 > ADAM23 > Cathepsin D. ABCA1, AKT1 and GATA3 all exhibited affinity scores for HPβCD that were within the lower micromolar range. It must be noted, however, that the *k_a_* scores for these proteins were at least 10-fold lower than that for SFRP1. Altogether, the data suggest that while HPβCD has potential to bind to all tested ligands, its most preferred target is SFRP1. To further investigate the effects of HPβCD on SFRP1 expression in MDA-MB-231 cells, immunofluorescent staining was performed. A 3.95-fold increase in SFRP1 expression was documented following a 24 h treatment period (Figure 6b,c). From this observation, it could be postulated that treatment with HPβCD stabilizes the tumour suppressor SFRP1 to reduce BC proliferation and aggressiveness.

## 4. Discussion

Despite several advancements in drug discovery, BC has been classified as an urgent global priority, presenting with a dismal 5-year relative cancer-specific survival rate, which is further exacerbated in patients diagnosed with TNBC [49]. This points to the poor efficacy of current treatment regimens, necessitating the investigation of novel avenues to combat this heterogenous disease [49]. Importantly, emerging experimental evidence highlights the pleotropic role of cholesterol in BC [50]. On this basis, the current study aimed to investigate the potential of a cholesterol-depletory agent (HPβCD) as a potentially promising anti-BC agent.

Historically, βCDs have been predominantly employed as excipients. This is attributed to the hydrophobic interior and hydrophilic exterior, which enhances solubility of poorly soluble drugs, consequently leading to increased bioavailability. Investigating HPβCD without any complex and as a stand-alone agent for diseases has recently garnered attention due to its cholesterol-depletory properties. HPβCD is being investigated currently as a strategy to treat Niemann–Pick disease type C. It successfully passed Phase 1 clinical trials in 2013 and received the FDA’s Breakthrough Therapy designation [43]. On this basis, Phase 2b/3 clinical trial efficacy tests are currently underway [31]. Despite success in Niemann–Pick Disease Type C, studies conducted on cancer are limited. To address this caveat and to explore the potential of HPβCD as an anticancer agent, the present study was conducted in two phases. In the first phase, the efficacy of HPβCD against BC was tested using primary screening assays to determine the effects on cell growth inhibition and apoptosis induction. The mechanism of cell death was then elucidated by investigating alterations to the cellular cholesterol profile and correlating this with gene expression studies along with drug interaction studies. In the second phase, HPβCD was administered to MF-1 mice xenografts to validate its efficacy against TNBC tumours and to further characterize its safety profile in mice. Previous studies with leukaemia [26] and atherosclerosis [51] have demonstrated the potential benefits associated with HPβCD use. Yokoo et al. [26] tested HPβCD across different leukaemia cell lines, and the reported IC_50_ values ranged from ~4 mM to ~10 mM across different time points up to 72 h, with the observed effects attributed to G_2/M_ mitotic cell-cycle arrest and apoptosis. Using similar concentrations, Zimmer et al. [41] reported a dose-dependent decrease in the overall cholesterol content following treatment with HPβCD in atherosclerosis. Varying concentrations were tested in the current study at three different time points (24 h, 48 h and 72 h). Both MCF-7 and MDA-MB-231 cancerous cell lines displayed IC_50_ values of 11.11 mM and 12.34 mM, respectively, thus confirming cell growth inhibition. This supports the results of the present study, where significant apoptotic induction, ~50%, was observed in MCF-7 and MDA-MB-231 cell lines. One of the key steps to apoptosis induction is the loss of MOMP. Mitochondria plays a crucial role in the signal transduction during apoptosis, it incites activation of the caspase family via the reduction of MOMP (Δ*ψ*m), which leads to the release of cytochrome *c* prior to the initiation of apoptosis. CyDs have been reported to lower Δψm, leading to the activation of Caspase-3/7 activity in a human oral squamous carcinoma cell line [24]. The present study also demonstrated similar results where MOMP-mediated apoptosis by HPβCD was established for MCF-7 and MDA-MB-231 cell lines at 92.1% and 78.1%, respectively, with the completion of the Caspase-3/7 assay yielding elevated levels (4-folds and 2.5-fold) in both the MCF-7 and MDA-MDA-231 cell lines, respectively, following treatment. This consequently confirmed apoptotic induction via the mitochondrial-mediated (intrinsic) pathway. Following this, quantification of the cellular cholesterol profile showed an overall reduction in cellular cholesterol content after treatment with various concentrations of HPβCD (1–50 mM). This was further supported by the cholesterol staining assay where free cholesterol and CE levels decreased by approximately 80% and 72%, respectively. The observed decrease in CEs is crucial given the important roles CEs play in facilitating cancer aggressiveness and drug resistance [52]. Importantly, a plethora of evidence exists demonstrating that lipid rafts play crucial roles in the signal transduction pathways along with receptor tyrosine kinases and glycosylphosphatidylinisotol (GPI)-anchored proteins [53,54]. Consequently, the involvement of lipid-raft-mediated cholesterol-depletion was also shown in the present study, with a 77% and 85% reduction reported in MCF-7 and MDA-MB-231 cells, respectively. These findings corroborate well with other studies, where in the K562 cell line (erythroleukemic cell line), following HPβCD treatment, p-Lyn (known to reside in lipid rafts) levels were greatly reduced [55]. Similarly, following the administration of HPβCD, TβR1 expression significantly decreased in BC cells leading authors to postulate that treating cells with HPβCD affected the distribution of TβR1 in lipid rafts [28]. These results further validate a positive correlation between HPβCD-mediated cholesterol-depletion in lipid rafts. To date, limited studies exist illustrating the effect of HPβCD on cholesterol metabolism. A study conducted in 2017 indicated that in cholesterol-loaded cells, HPβCD had the potential to expand the active cholesterol pool, being the first study documenting that SREBP1 levels were reduced following HPβCD treatment [56]. Contrary to this, in MDA-MB-231 cells, a dose-dependent increase in the expression of HMGCR is reported, with reduced expression of cholesterol efflux proteins ABCA1 and ABCG1 following treatment with HPβCD [57]. This consequently necessitates further mechanistic investigations pertaining to exactly how HPβCD modifies the cellular cholesterol composition in various cancer cell types. From this, it can be stated that treatment with cholesterol-depleting agents like CyDs elicits an overall depletion in cholesterol levels, which consequently inhibits growth of cells via apoptosis induction. Interestingly, the initiation of apoptosis is limited to only cholesterol-extracting CyDs, such as MβCD and HPβCD, which may be attributed to the disruption of cholesterol-rich microdomains due to the depletion of cholesterol from cellular membranes [53,54].

**Figure 7:**
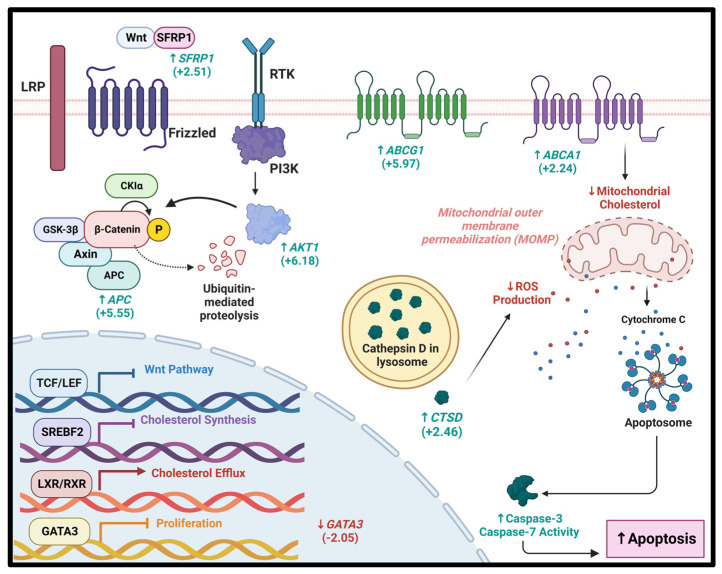
Graphical summary of hypothetical molecular mechanisms at play in HPβCD-mediated apoptosis. Treatment with HPβCD potentially abrogates genes involved in cholesterol synthesis while facilitating cholesterol efflux, consequently reducing the cellular cholesterol profile of BC cells. Moreover, HPβCD binds to SFRP1, a negative regulator of the Wnt pathway, possibly leading to ubiquitin-mediated proteolysis of the nuclear transcription factor β-catenin. Additionally, binding of HPβCD to AKT1 could potentially exacerbate this, thereby inhibiting the binding of β-catenin to TCF/LEF binding sites. This ultimately leads to Wnt pathway inhibition. Furthermore, the binding of HPβCD to GATA3 (a zinc finger transcription factor) could potentially reduce proliferation. Interestingly, the ABCA1-mediated decrease of mitochondrial membrane cholesterol facilitates the release of mitochondrial-derived cell-death-promoting molecules, such as Cytochrome C, leading to formation of the apoptosome. This leads to subsequent activation of the caspase cascade with increased activity in effector activity (Caspase 3/7). This mechanism, coupled with a Cathepsin-D (CTSD)-mediated decrease in ROS levels, could serve as a crucial means by which HPβCD induces mitochondrial-mediated apoptosis. (Values represent fold change from MDA-MB-231 arrays expression data in respective genes – Green (Up Arrow) = Upregulated; Red (Down Arrow) = Downregulated) (Created using Bioreder.com (accessed on 10 May 2023)).

To identify specific molecular targets of HPβCD, RT^2^ Profiler PCR Arrays were employed to investigate the changes in gene expression profiles of 168 genes corresponding to genes involved in cancer-drug resistance, and cholesterol and lipoprotein metabolism. Cytoscape analysis was subsequently completed to understand the pathway dynamics in HPβCD-treated cells. Different gene expression profiles were observed in MCF-7 vs. MDA-MB-231 cells, which demonstrate that at the molecular level, HPβCD acts in a cell-line-specific manner, thus emphasizing the importance of further investigations into the various BC subtypes. Based on the gene expression profiles, their relevance to cancer and cholesterol-pathways, six proteins were shortlisted for HPβCD binding studies using SPR. We demonstrate for the first time that Secreted frizzled-related protein 1 (SFRP1) displayed the greatest binding affinity towards HPβCD. Importantly, SFRP1’s role as a tumour suppressor has been elucidated across a plethora of literature. *SFRP1* is a known antagonist of the Wnt pathway and inhibits *WNT*-dependent transcription, leading to a decrease in the intracellular level of β-catenin (Wnt activator) [58] Importantly, a deregulated Wnt signalling pathway is implicated in cancer progression, stemness, multidrug resistance, and immune escape [59]. In BC, approximately 50% of patients present with hyperactivation of the Wnt pathway, which is linked to reduced overall survival [60]. Interestingly, hypermethylation of the promoter regions of the gene encoding for SFRP1 reduces *SFRP1* expression in 80% of all BC cases [61]. This is further validated by a recent study indicating that *SFRP1* expression is negatively associated with ER, PR, and HER 2 statuses. Low SFRP1 expression was further linked to significantly poorer survival and therapy resistance [62]. This is supported by evidence indicating that SFRP1-postive BC tumours present with lower tumour stage following neoadjuvant therapy [61]. Additionally, the downregulation of *SFRP1* in both human and mice (*SFRP1^−/−^)* is responsible for increased oestrogen-induced response and hyperplasia [63]. A genome-wide association study reported that, in the *SFRP1*-modulated gene interaction network, 27 genes were modulated in a cluster involved in oestrogen stimulus response [64]. We hypothesize that HPβCD quite likely can bind to SFRP1 and accentuate its tumour suppression characteristics. This further potentiates the use of SFRP1 as a diagnostic biomarker to predict patient prognosis, which should be further explored as a therapeutic target in BC. In addition to SFRP1, HPβCD possibly targets several arms of the Wnt signalling pathway through its binding to and upregulation of AKT1 (Figure 7). Aside from the AKT1’s well-documented role in promoting cancer survival, emerging evidence implicates AKT1 in reducing the expression and impeding the nuclear accumulation of β-catenin, consequently reducing invasive potential [65,66]. Based on this, in-depth investigations into multiple roles of AKT1 are needed to delineate the multifaceted role it plays in tumorigenesis. Interestingly, the binding of HPβCD to GATA3 reduced GATA3 expression. Conventionally, GATA3 impedes tumorigenesis, epithelial-mesenchymal transition (EMT) and metastasis [67]. Interestingly, GATA3 has proved useful in differentiating between primary and metastatic tumours in patients with a history of BC regardless of molecular subtype [68]. Despite GATA3’s known role as a tumour suppressor and diagnostic biomarker, the HPβCD-mediated suppression of GATA3 could prove beneficial. This is supported by recent studies indicating that GATA3 is mutated in a high frequency of TNBCs, making it a driver of BC progression by conferring cells with a proliferative advantage [69,70]. This corroborates well with a study conducted by Gulbache et al., in which ER^−^/GATA-3^+^ tumours had worse breast cancer survival (BCS) (*p* = 0.02) and a trend for worse overall survival (OS) (*p* = 0.05) compared to ER^−^/GATA-3^−^ tumours [71]. Based on this conflicting evidence, the true role of GATA3 in BC requires further investigation. In like manner, HPβCD binding to ADAM23 potentially facilitates tumour suppression due to its adhesive function. This is important given that this gene is often hypermethylated in BC, leading to a ninefold increased risk of developing distant metastases, subsequently translating to lower survival rates [72].

Intriguingly, HPβCD displayed a high binding affinity to the major cholesterol efflux protein ABCA1. The tumour suppressive effects of ABCA1 could be attributed to its role in reducing mitochondrial cholesterol levels under conditions of increased cholesterol synthesis [73]. HPβCD binding to ABCA1 could thus prove beneficial by facilitating a reduction in mitochondrial cholesterol, increasing mitochondrial membrane permeability consequently facilitating the release of cytochrome C [73]. Furthermore, HPβCD binding to Cathepsin D may justify the relatively low levels of ROS production in cells undergoing apoptosis. This can be corroborated by evidence indicating a novel role of Cathepsin D in maintaining lysosomal membrane integrity and redox balance [74]. Based on this, it can be potentially hypothesised that the effect of HPβCD, principally on these proteins (ABCA1 and Cathepsin D), ultimately manifests in mitochondrial-mediated apoptosis.

Previous studies with HPβCD for leukaemia [26] and atherosclerosis [41] provided valuable insights concerning the dosage requirements and the mode of administration. IP injections of HPβCD were seen to significantly improve the survival in leukaemia mouse models and administration of HPβCD was seen to prolong their survival [26]. It was also observed in this study that HPβCD-treated mice survived longer than vehicle-treated mice, and the overall survival of mice that received 695.5 mg/kg–2086.5 mg/kg HPβCD was substantially higher than that of vehicle-treated mice, thus suggesting HPβCD is quite non-toxic to the normal physiology of living animals. Subsequently, these studies formed the basis of our in vivo study, and we successfully achieved significant regression in tumour sizes with minimal toxicity. These studies are corroborated by findings in other studies documenting no significant adverse effects reported on liver function ((AST), (ALT) and alkaline phosphatase (ALP) activity) and renal function (BUN and CRE) when using doses of 2000 mg/kg [75]. Some studies even went to the extent of administering 4000 mg/kg to mice. Interestingly, serum AST and ALT were reduced at this dosage [76]. Furthermore, administering 40,000 mg/kg HPβCD to a patient provided a good fit to the theoretical pharmacokinetic parameters estimated [76]. In a study conducted in rats, 4500 mg/kg of 45% HPβCD, when administered for one month, resulted in minor haematological changes and increased plasma liver enzyme levels. There was no evidence of any histopathological changes, even at the highest oral dose, indicating the toxicologically benign nature of HPβCD [29]. In general, administration of HPβCD via intravenous infusion induced some minor clinical observations as well as biochemical and histopathological changes. The target organs were the lungs, where there was an increase in macrophage infiltration in the liver and kidneys (in some studies histopathological findings were noted in both organs). In studies that looked for a dose response, no effect dose level was achieved, and where reversibility was studied, all findings were reversible [29]. Importantly, further validation in human studies indicate that prolonged treatment with HPβCD is well tolerated in humans with minimal safety issues documented [77].

## 5. Conclusions

In conclusion, this study proposes an innovative strategy for the treatment of ER^+^ and TNBC. We have demonstrated in vitro that HPβCD possesses capabilities to block cancer cell proliferation by altering the MOMP, consequently inducing apoptosis via the depletion of total cholesterol, CEs, and the disruption of lipid rafts. In the in vivo mice xenograft study, no liver toxicity was displayed while significantly reducing tumour sizes. It was further demonstrated that HPβCD induces molecular level changes in a cell-line-specific manner by affecting expression patterns of several genes involved in BC and cholesterol signalling pathways and binds principally to SFRP1 to impart its potential anticancer functions. In addition to the listed strengths, some inherent limitations are present, namely the usage of basic imaging instrumentation and lack of other protein-related assays that could have been performed to validate gene expression arrays. The combination of HPβCD with chemotherapeutic drugs should also be further tested, which is currently underway in our laboratory on colorectal and pancreatic cancer cell lines as well as xenograft mice models.

Nevertheless, findings from the current study provide us with insightful and novel information potentiating the use of HPβCD as a promising anti-breast cancer agent with limited known side-effects, which warrants further testing in a Phase IIa human clinical trial in BC patients.

## 6. Patent

HPβCD for use in the treatment of breast cancer. PCT patent application no: PCT/IN2020/050651. 28 January 2020. (National phase filing completed, August 2021).

## Figures and Tables

**Table 1:** Kinetics data for the interaction of HPβCD with human recombinant proteins.

Ligand	*K_a_* (1/M*s) ± est. Error	*K_d_* (1/s) ± est. Error	*K_D_* (M) ± est. Error	X^2^
ABCA1	3.55 (±0.003) × 10^+6^	6.27 (±0.012) × 10^−2^	1.77 (±0.46) × 10^−8^	1.45
ADAM23	3.73 (±0.27) × 10^+5^	7.58 (±0.32) × 10^−2^	2.03 (±0.15) × 10^−7^	7.19
AKT1	3.98 (±0.64) × 10^+6^	7.45 (±0.11) × 10^−2^	1.87 (±0.57) × 10^−8^	2.41
GATA3	6.57 (±0.39) × 10^+5^	5.92 (±0.36) × 10^−2^	9.00 (± 0.59) × 10^−8^	1.09
Cathepsin D	4.40 (±0.14) × 10^+5^	5.63 (±0.14) × 10^−2^	1.28 (±0.44) × 10^−7^	4.0
SFRP1	1.03 (±0.51) × 10^+7^	8.12 (±0.34) × 10^−2^	7.91 (±0.72) × 10^−9^	2.23

## Data Availability

The data presented in this study are available in this article and Supplementary Materials.

## Supplementary Materials

The following supporting information can be downloaded at: https://www.mdpi.com/article/10.3390/cancers15102828/s1, Figures S1–S12 and Table S1: 2-hydroxypropyl-β-cyclodextrin (HPβCD) as a potential therapeutic agent for breast cancer.
