# Supplementary material for: 2-Hydroxypropyl-β-cyclodextrin (HPβCD) as a Potential Therapeutic Agent for Breast Cancer
Source: Cancers (Basel). 2023 May 18;15(10):2828. doi: 10.3390/cancers15102828 (PMC10216648; doi:10.3390/cancers15102828)
Supplement: Supplementary file 1 [file cancers-15-02828-s001.zip › cancers-2359807-supplementary.pdf]

Supplementary Materials

2-Hydroxypropyl-β-cyclodextrin (HPβCD) as a Potential Therapeutic Agent for Breast Cancer

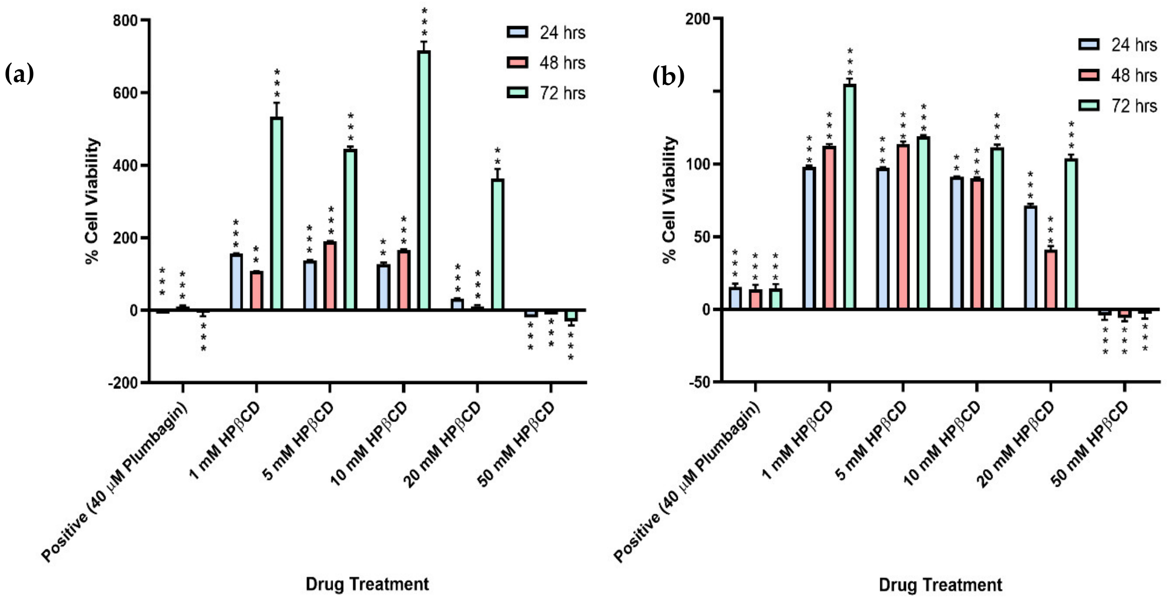

Figure S1. Graph comparing the percentage growth inhibition of **(a)** MRC-5 **(b)** HEK-293 cells at selected concentrations of HPβCD at 1 mM, 5 mM, 10 mM, 20 mM and 50 mM relative to the untreated control. PL (40 μM) was used as a positive control. A one-way ANOVA test was complete for statistical analysis. A Bonferroni post-hoc test was employed for pairwise analysis comparing treated groups to the untreated groups. Data represents mean ± standard deviation S.D. (n=3) from raw data, where *p<0.05, **p<0.01 and ***p<0.001 significant difference to untreated control. Independent experiments were run at least 3 times.

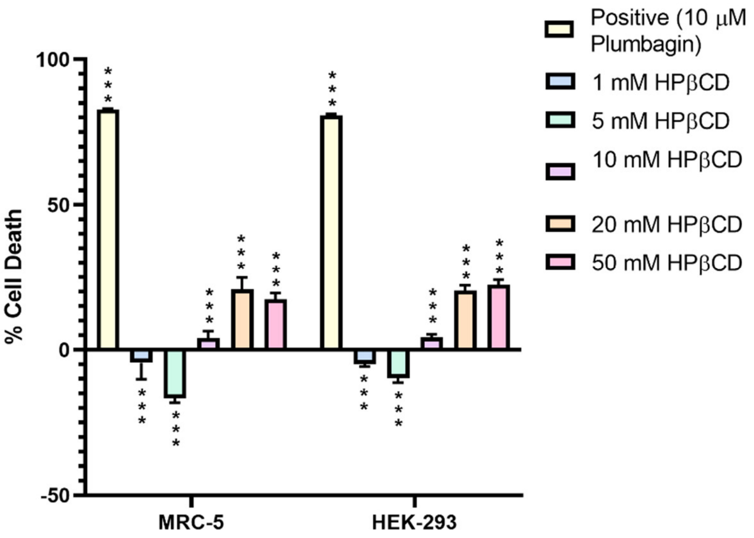

Figure S2. Graph comparing the percentage of apoptosis in MRC-5 and HEK-293 cells at selected concentrations of HPβCD at 1 mM, 5 mM, 10 mM, 20 mM, 50 mM and negative samples. PL (10µM) was used as a positive control. A one-way ANOVA test was complete for statistical analysis. A Bonferroni post-hoc test was employed for pairwise analysis comparing treated groups to the untreated groups. Data represents mean ± standard deviation S.D. (n=3) from raw data, where *p<0.05, **p<0.01 and ***p<0.001 significant difference to untreated control. Independent experiments were run at least 3 times.

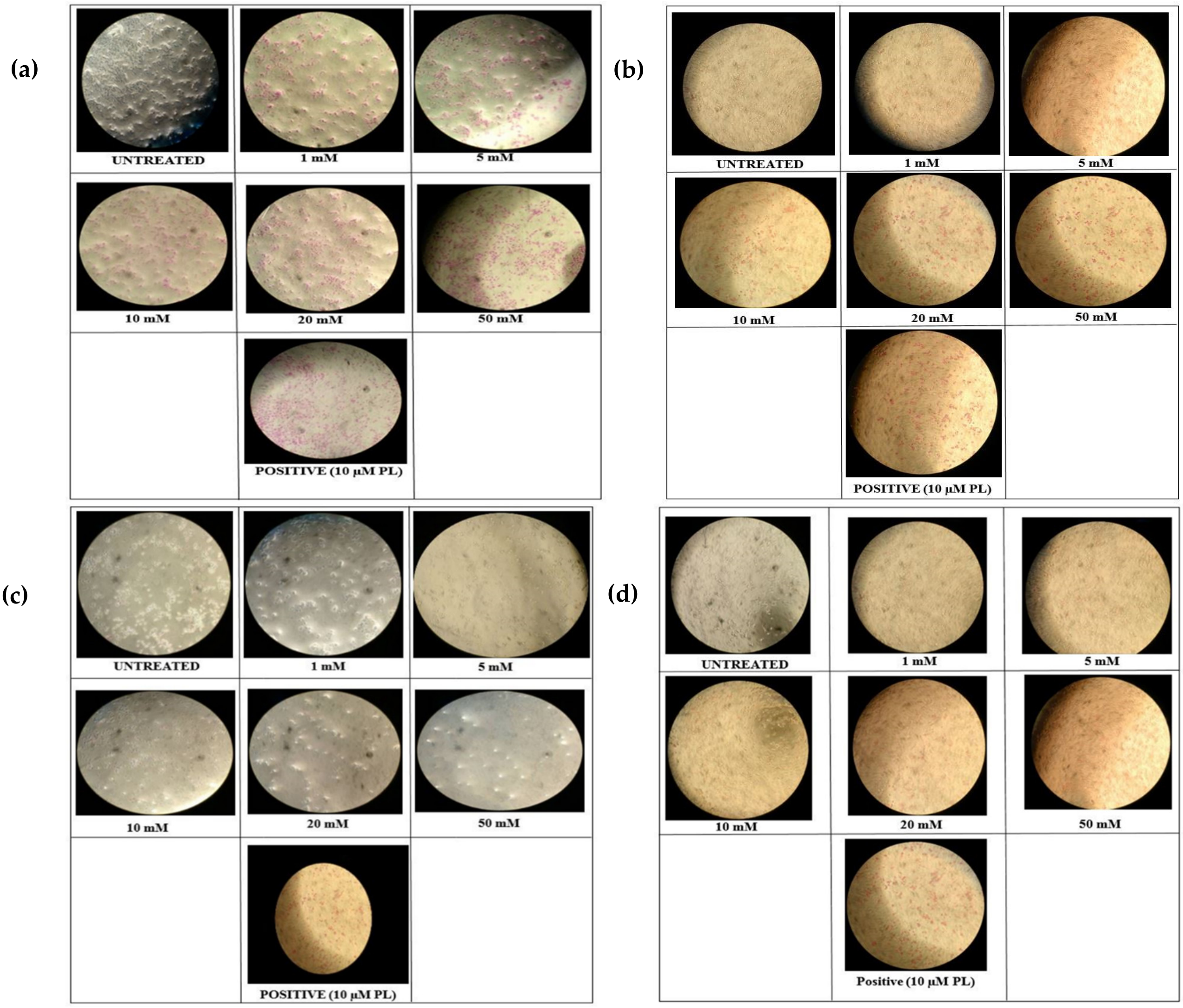

Figure S3. Light microscope images showing the amount of apoptosis in **(a)** MCF-7 **(b)** MDA-MB-231-**(c)** MRC-5 and **(d)** HEK-293 cells at selected concentrations of HPβCD at 1 mM, 5 mM, 10 mM, 20 mM, 50 mM and untreated samples. PL was used as a positive control. Images were captured at 10x magnification.

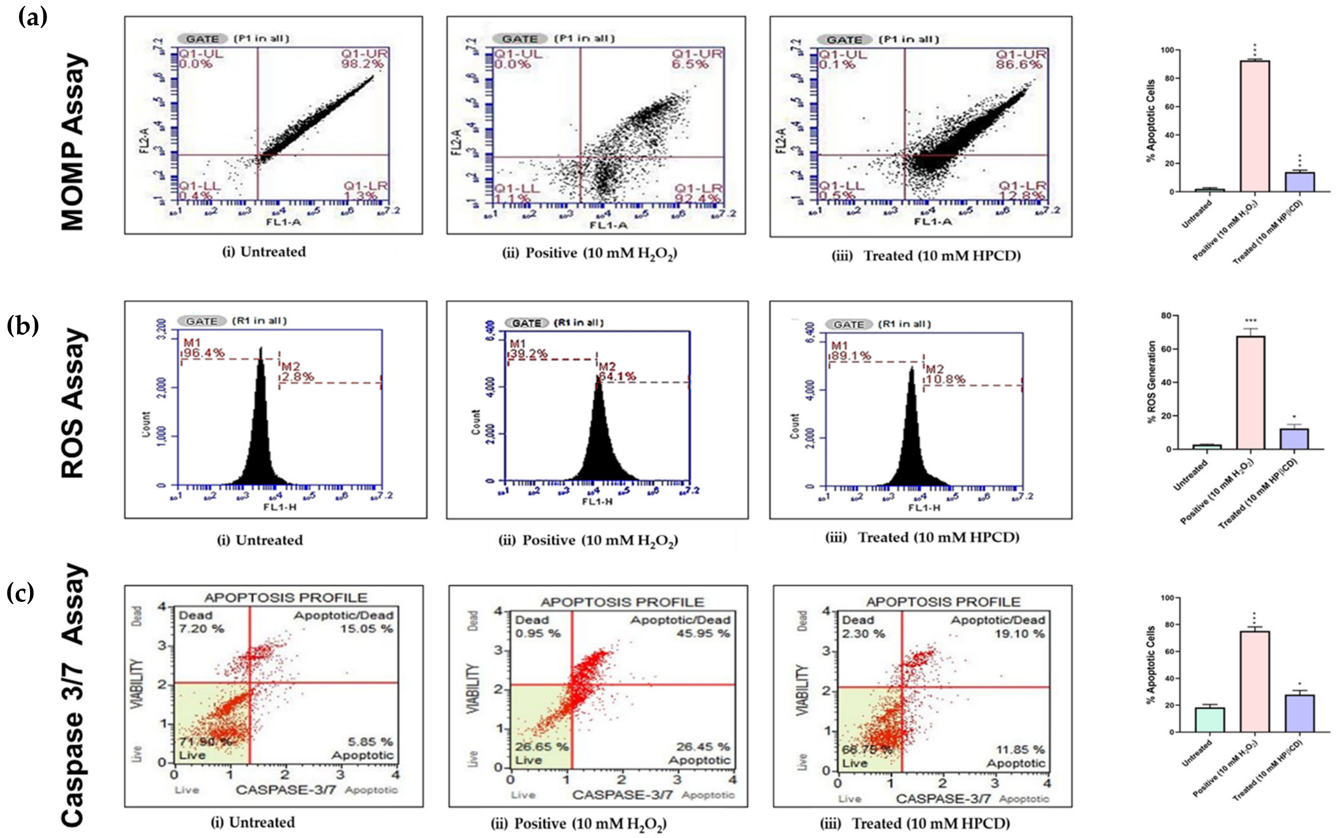

Figure S4. (a) MOMP plots for MRC-5 cells. (i): Loss of mitochondrial membrane potential in untreated cells, (ii): Loss of mitochondrial membrane potential in positive sample (10 mM H_2O_2) and (iii): Loss of mitochondrial membrane potential at 10 mM HPβCD. **(b)** ROS generation in MRC-5 cells. (i): ROS generation in untreated cells (2.8 %), (ii): ROS generation in positive sample (10 mM H_2O_2) (64.1 %) and (iii): ROS generation at 10 mM HPβCD (10.8 %) **(c)** Caspase-3/7 profile in HEK-293 cells. (i): Total apoptotic/live cells generation in untreated cells, Total Live – 71.90 %, Total apoptotic- 20.9 % (ii): Total apoptotic/live cells generation in positive sample (10 mM H_2O_2), Total Apoptotic – 72.40 % and (iii): Total apoptotic/live cells generation in treated (10 mM HPβCD) cells, Total Live- 66.75%, Total apoptotic- 30.95 %. A one-way ANOVA test was complete for statistical analysis. A Bonferroni post-hoc test was employed for pairwise analysis comparing treated groups to the untreated groups. Data represents mean ± standard deviation S.D. (n=3) from raw data, where *$p<0.05$, **$p<0.01$ and ***$p<0.001$ significant difference to untreated control. In-dependent experiments were run at least 3 times.

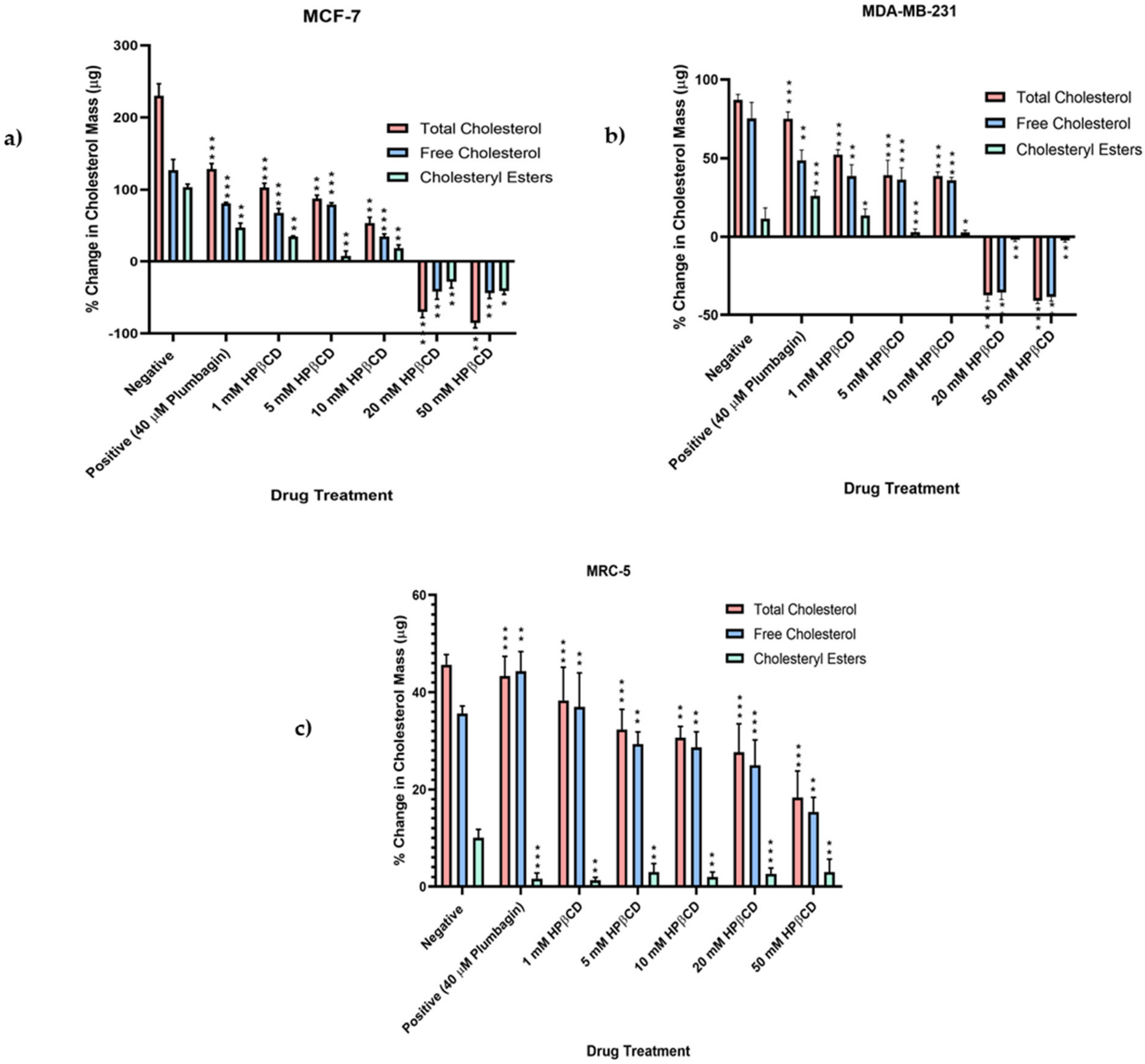

Figure S5. Representative graph comparing the percentage change in mass of total, free and esterified cholesterol levels in **(a)** MCF-7 (**b**) MDA-MB-231 and **(C)** MRC-5 cells at selected concentrations of HPβCD at 1 mM, 5 mM, 10 mM, 20 mM, 50 mM and negative samples. PL was used as a positive control. Total cholesterol concentration is determined by a coupled enzyme assay, which results in a colorimetric (570 nm)/fluorometric (λex = 535/λem = 587 nm) product, proportional to the amount of cholesterol present. Due to the colorimetric nature of the assay, variability in the data was evident which led to the use of this assay as a preliminary assay to validate results obtained in viability and cytotoxicity assays. This result was further supplemented with cholesterol staining. A one-way ANOVA test was complete for statistical analysis. A Bonferroni post-hoc test was employed for pairwise analysis comparing treated groups to the untreated groups. Data represents mean ± standard deviation S.D. (n=3) from raw data, where *p<0.05, **p<0.01 and ***p<0.001 significant difference to untreated control.

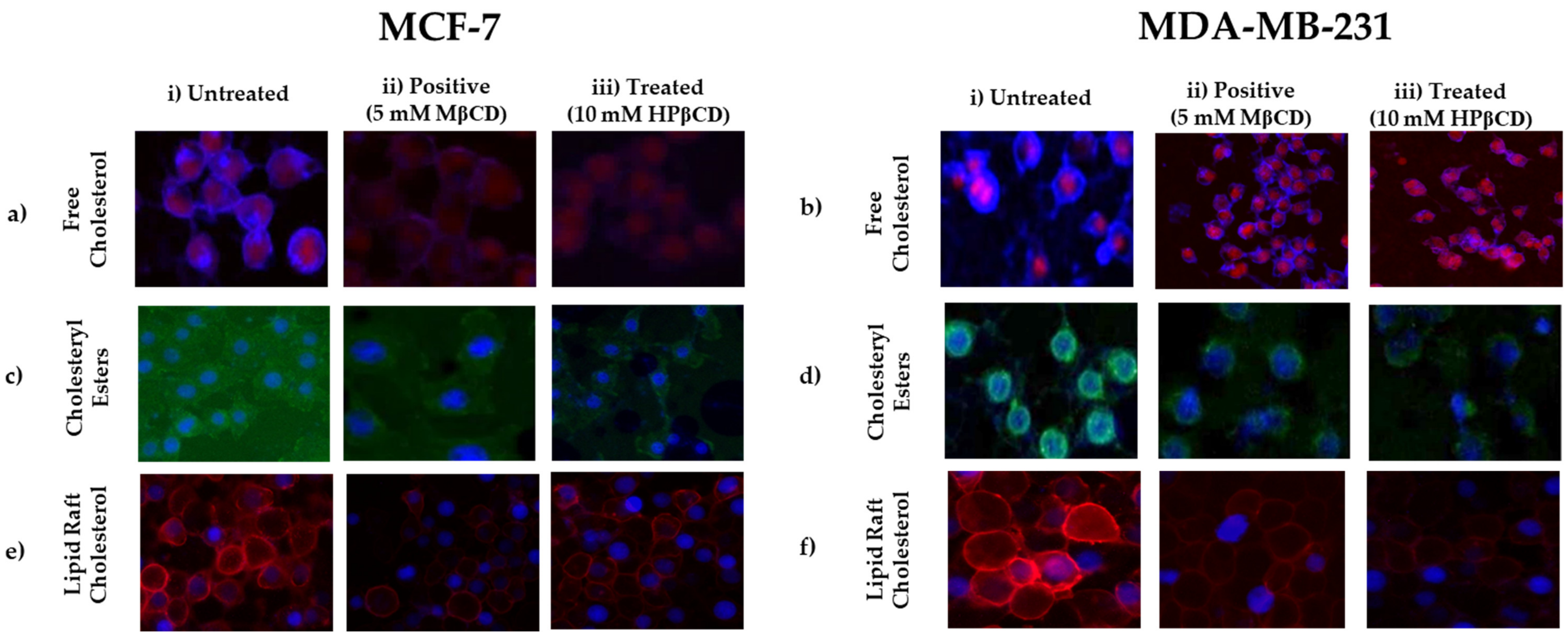

Figure S6. Filipin staining of **(a)** MCF-7 **(b)** MDA-MB-231 cells to determine free cholesterol content. Lipid droplet staining of **(c)** MCF-7 **(d)** MDA-MB-231 cells to determine cholesteryl ester content. Lipid raft staining of **(e)** MCF-7 **(f)** MDA-MB-231 cells to determine lipid raft cholesterol content. Comparison of staining in untreated, positive (5 mM MβCD) and treated (10 mM HPβCD). Images were captured using the Floid™ Cell Imaging System followed by analysis using the Image J software. .

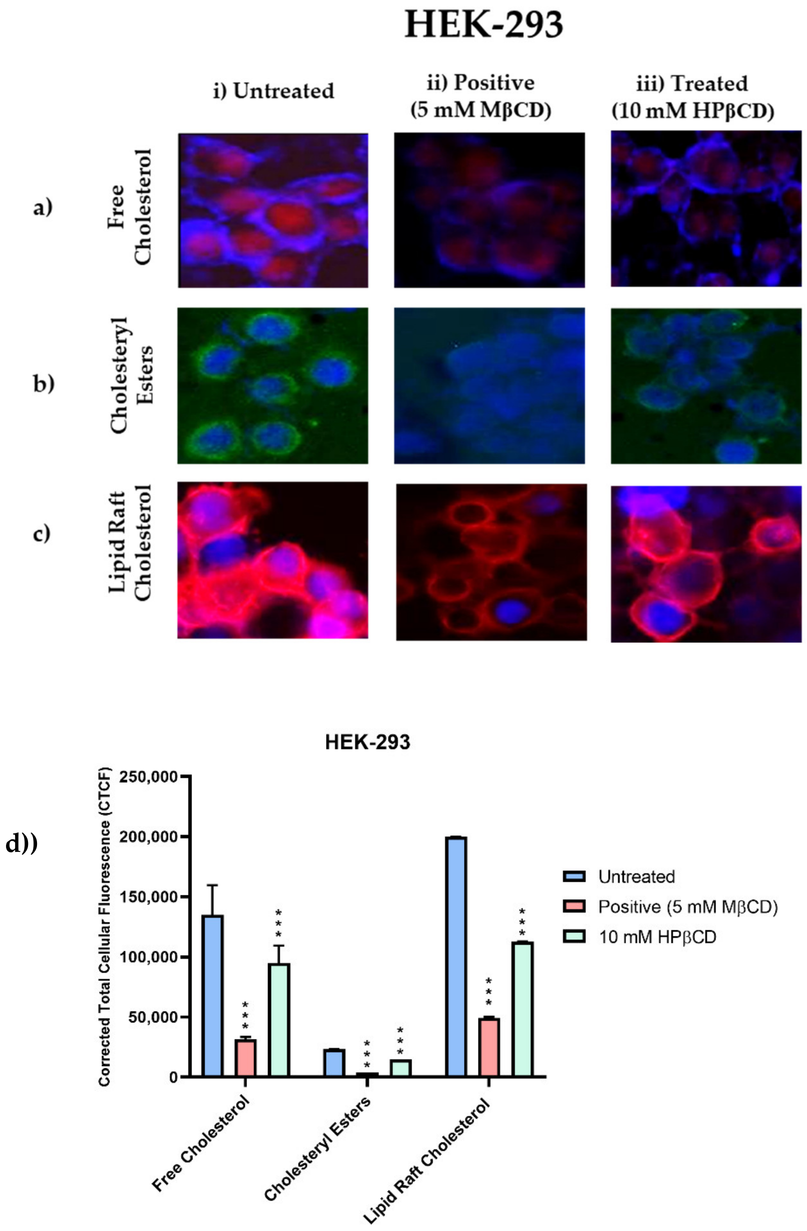

Figure S7. (a) Filipin staining of HEK-293 cells to determine free cholesterol content. **(b)** Lipid droplet staining of HEK-293 cells to determine cholesteryl ester content. **(c)** Lipid raft staining of HEK-293 cells to determine lipid raft cholesterol content. Comparison of staining in untreated, positive (5 mM MβCD) and treated (10 mM HPβCD). Images were captured using the Floid™ Cell Imaging System followed by analysis using the Image J software. A one-way ANOVA test was complete for statistical analysis. A Bonferroni post-hoc test was employed for pairwise analysis comparing treated groups to the untreated groups. Data represents mean ± standard deviation S.D. (n=3) from raw data, where $^{*}p<0.05$, $^{**}p<0.01$ and $^{***}p<0.001$ significant difference to untreated control. Independent experiments were run at least 3 times.

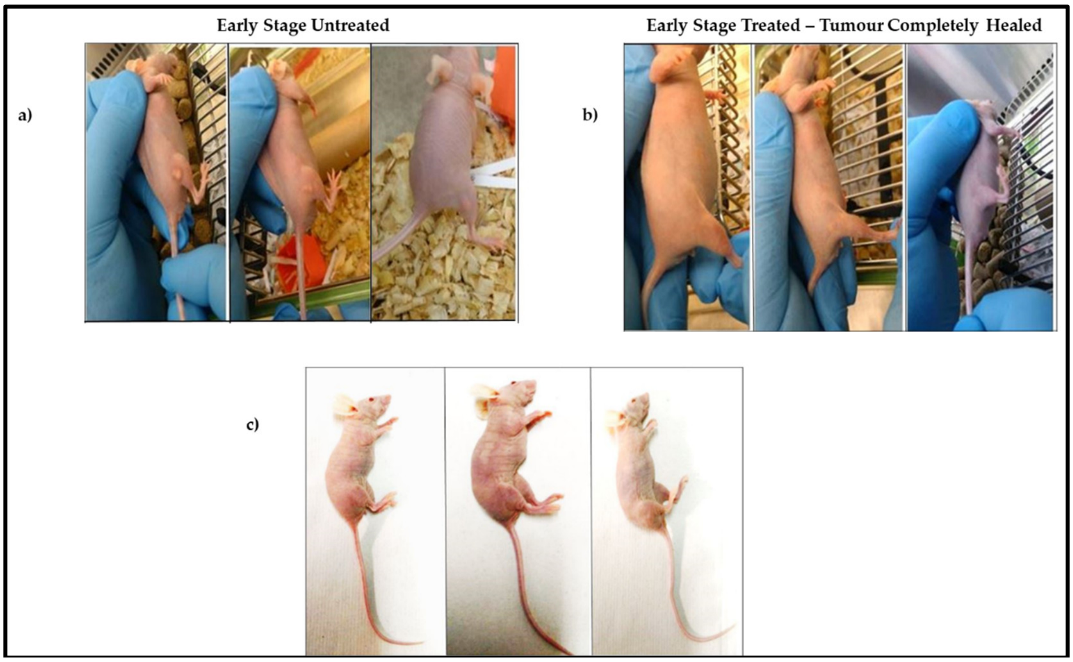

Figure S8. (a) Mice images post euthanization showing tumour sizes in untreated group at an early stage, injected with MDA-MB-231 cells. **(b)** Mice images post euthanisation showing tumour sizes in treated group at an early stage, injected with MDA-MB-231 cells and treated with HPβCD (3000 mg/kg b.w.) for 10 doses. Results show complete healing of tumour (100%) compared to the untreated group. **(c)** All three healed mice (No. 12, 13 and 14) tested for relapse of TNBC (MDA-MB-231) tumour for a period of 4 weeks. Results show no relapse at all.

(a)

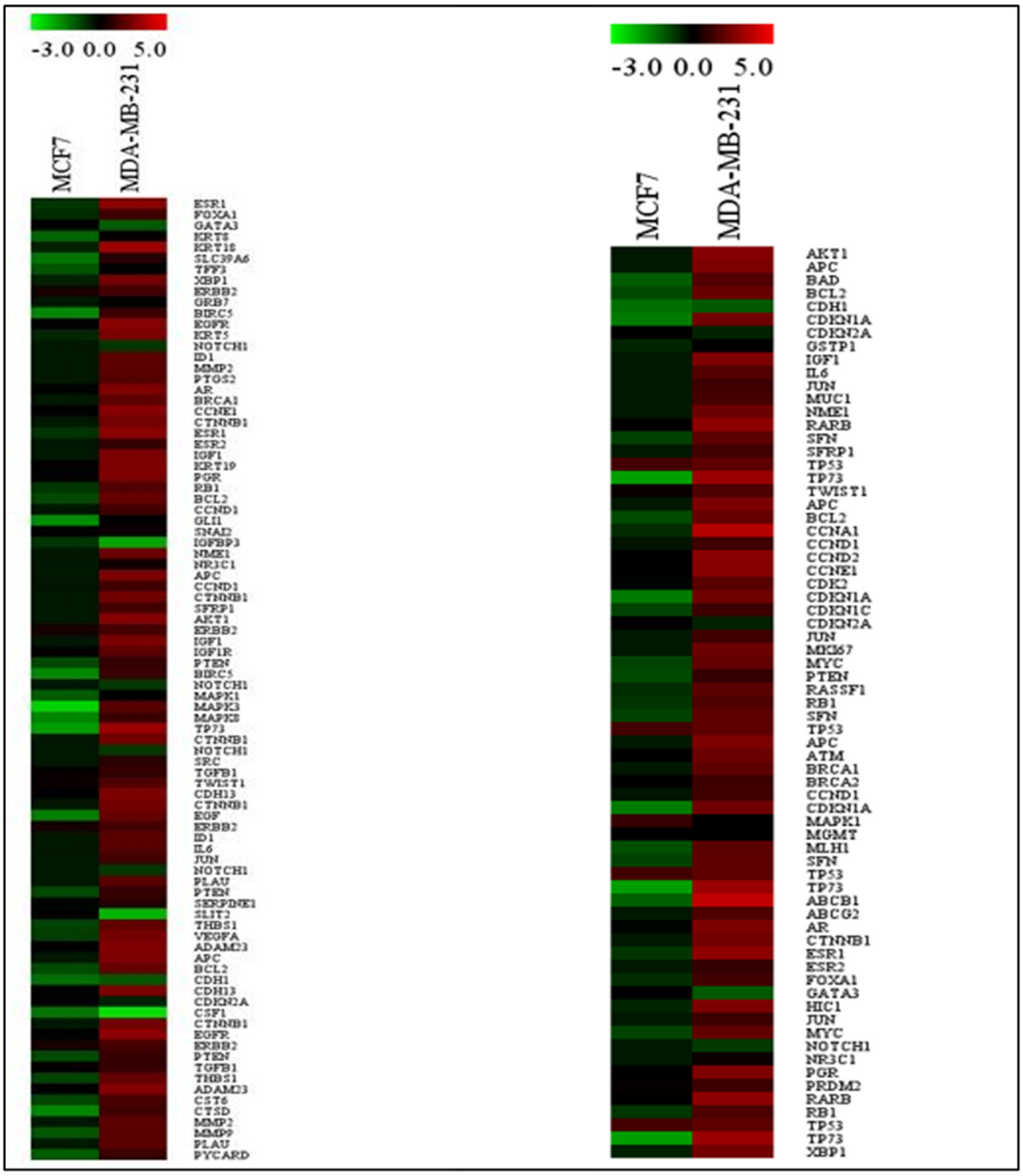
-3.0 0.0 5.0
MCF7
MDA-MB-231
ESR1
FOXA1
GATA3
KRT8
KRT18
SLC39A6
TFF3
XBP1
ERBB2
GRB7
BIRC5
EGFR
KRT5
NOTCH1
ID1
MMP2
PTGS2
AR
BRCA1
CCNE1
CTNNB1
ESR1
ESR2
IGF1
KRT19
PGR
RB1
BCL2
CCND1
GLI1
SNAI2
IGFBP3
NME1
NR3C1
APC
CCND1
CTNNB1
SFRP1
AKT1
ERBB2
IGF1
IGF1R
PTEN
BIRC5
NOTCH1
MAPK1
MAPK3
MAPK8
TP73
CTNNB1
NOTCH1
SRC
TGFB1
TWIST1
CDH13
CTNNB1
EGF
ERBB2
ID1
IL6
JUN
NOTCH1
PLAU
PTEN
SERPINE1
SLIT2
THBS1
VEGFA
ADAM23
APC
BCL2
CDH1
CDH13
CDKN2A
CSF1
CTNNB1
EGFR
ERBB2
PTEN
TGFB1
THBS1
ADAM23
CST6
CTSD
MMP2
MMP9
PLAU
PYCARD
-3.0 0.0 5.0
MCF7
MDA-MB-231
AKT1
APC
BAD
BCL2
CDH1
CDKN1A
CDKN2A
GSTP1
IGF1
IL6
JUN
MUC1
NME1
RARB
SFN
SFRP1
TP53
TP73
TWIST1
APC
BCL2
CCNA1
CCND1
CCND2
CCNE1
CDK2
CDKN1A
CDKN1C
CDKN2A
JUN
MKI67
MYC
PTEN
RASSF1
RB1
SFN
TP53
APC
ATM
BRCA1
BRCA2
CCND1
CDKN1A
MAPK1
MGMT
MLH1
SFN
TP53
TP73
ABCB1
ABCG2
AR
CTNNB1
ESR1
ESR2
FOXA1
GATA3
HIC1
JUN
MYC
NOTCH1
NR3C1
PGR
PRDM2
RARB
RB1
TP53
TP73
XBP1

(b)

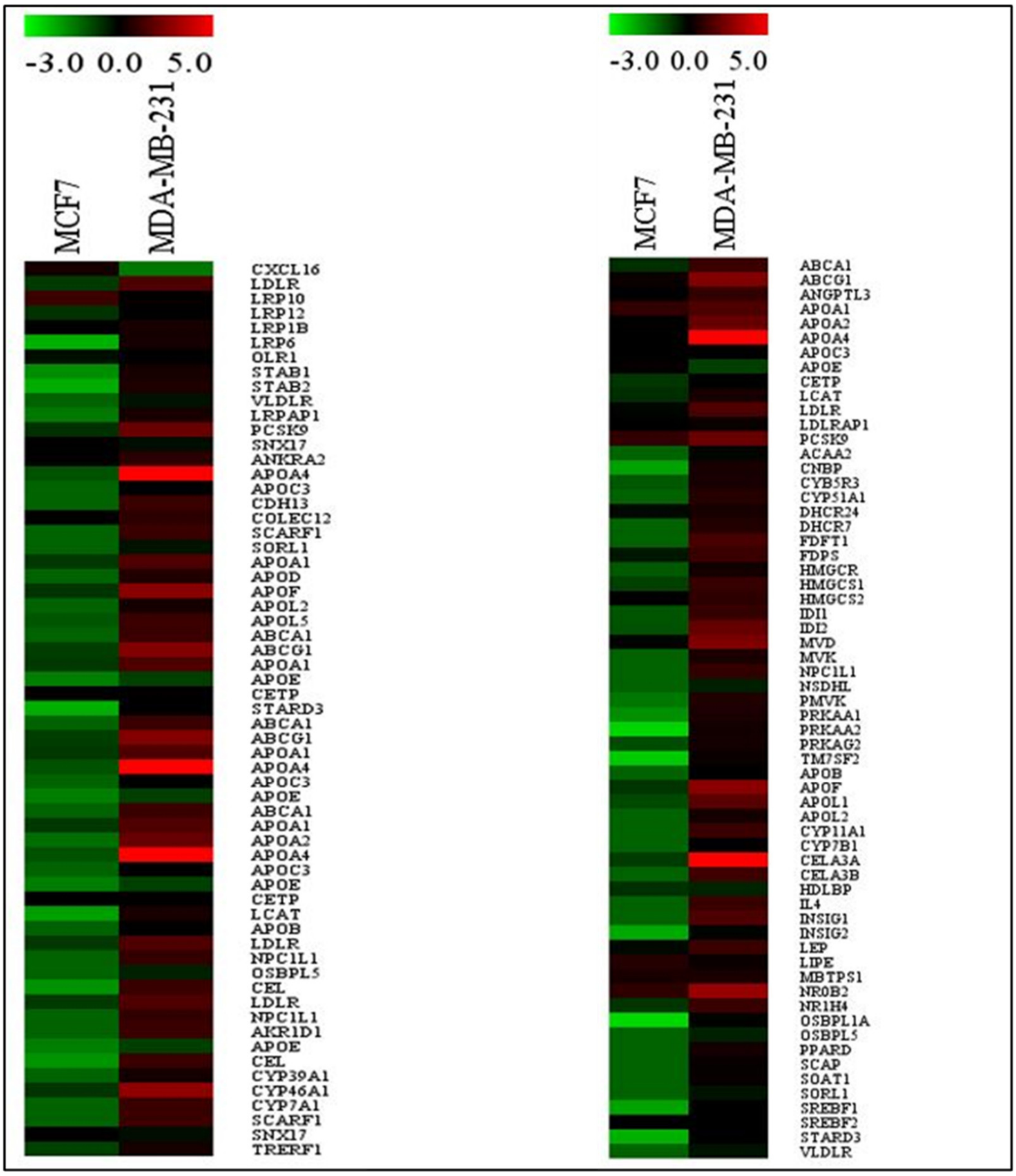

Figure S9. (a) List of genes up (red) and down-regulated (green) in human breast cancer signalling following treatment with 10 mM HPβCD in MCF-7 and MDA-MB-231 cell lines. Log2 was utilised to normalise the data against 1 to facilitate ease of interpretation. Cells were coloured according to fold Log2 fold change (control/ HPβCD treated cells) with the intensity of colour corresponding to the degree of fold change, **(b)** List of genes up (red) and down-regulated (green) in human lipoprotein signalling following treatment with 10 mM HPβCD in MCF-7 and MDA-MB-231 cell lines. Log2 was utilised to normalise the data against 1 to facilitate ease of interpretation. Cells were coloured according to Log2 fold change (control/ HPβCD treated cells) with the intensity of colour corresponding to the degree of fold change.

(a)

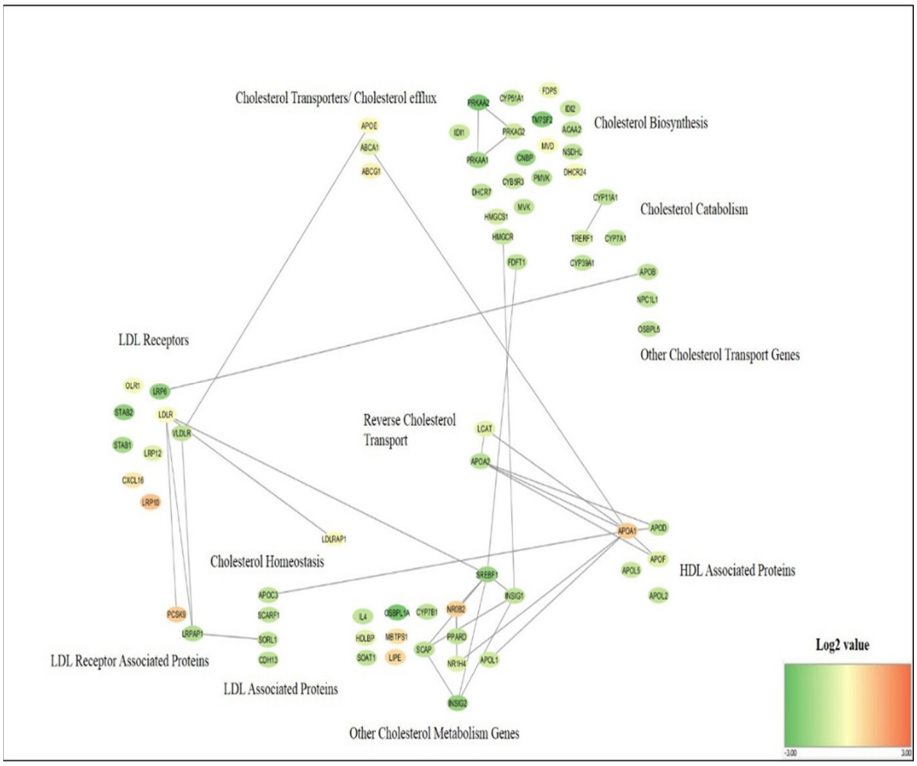

(b)

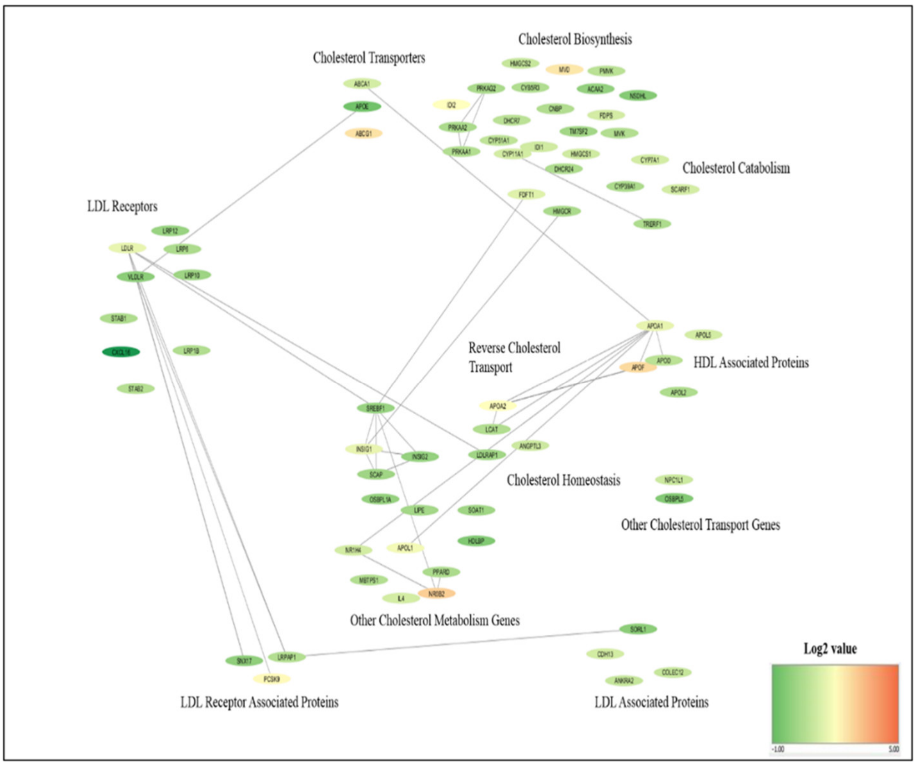

Figure S10. A network map of genes involved in human lipoprotein signaling in **(a)** MCF-7 cells **(b)** MDA-MB-231 following 10 mM HPβCD was created using Cytoscape. Nodes (genes) were colored according to Log_2 fold change (Untreated/treated MCF-7 and MDA-MB-231 cells) with the intensity of color corresponding to the degree of fold change. Potential gene interactions (links; coloured in grey) were generated using OmicsNet and visualized in Cytoscape. Genes were grouped according to the pathway they act in, demonstrating several intra-pathway and inter-pathway interactions. Green to orange represents low to high levels of expression.

(a)

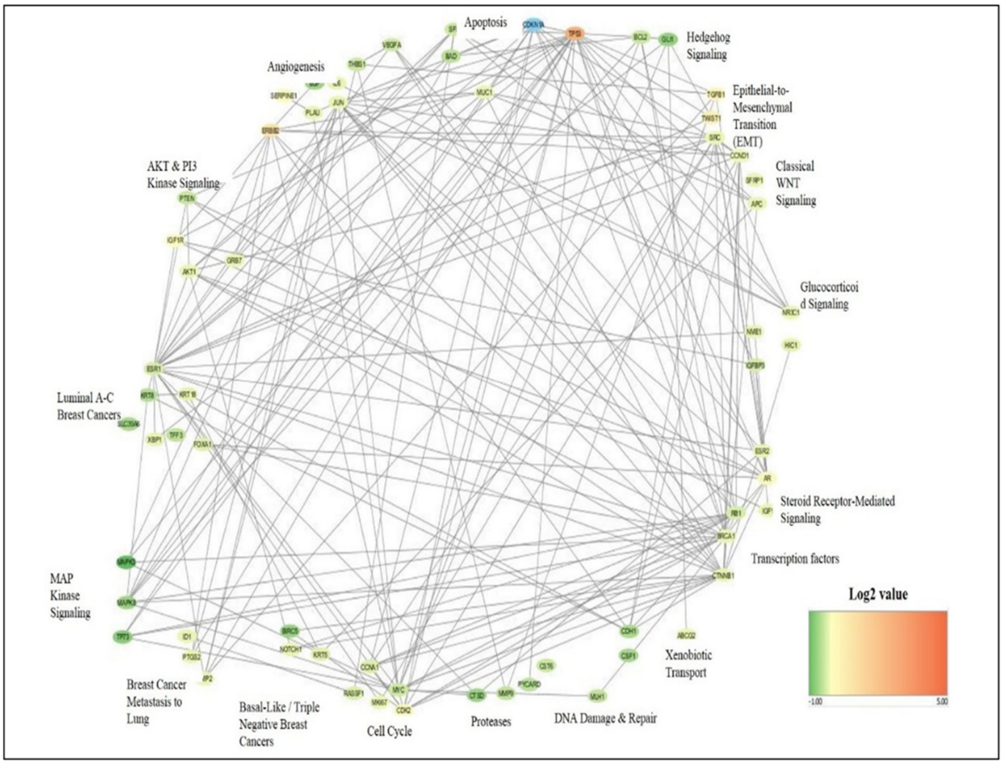

(b)

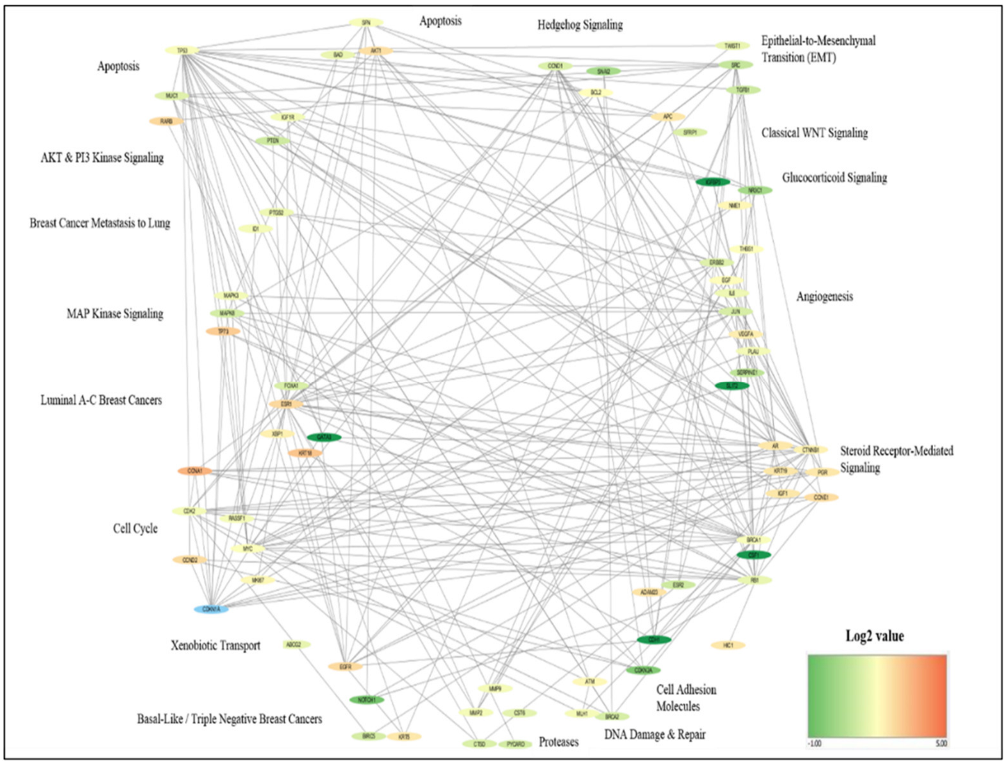

Figure S11. A network map of genes involved in human breast cancer **(a)** MCF-7 and **(b)** MDA-MB-231 cells following 10 mM HPβCD was created using Cytoscape. Nodes (genes) were colored according to Log_2 fold change (control/treated MCF-7 and MDA-MB-231 cells) with the intensity of color corresponding to the degree of fold change. Potential gene interactions (links;

coloured in grey) were generated using OmicsNet and visualized in Cytoscape. Genes were grouped according to the pathway they act in, demonstrating several intra-pathway and inter-pathway interactions. Green to orange represents low to high levels of expression.

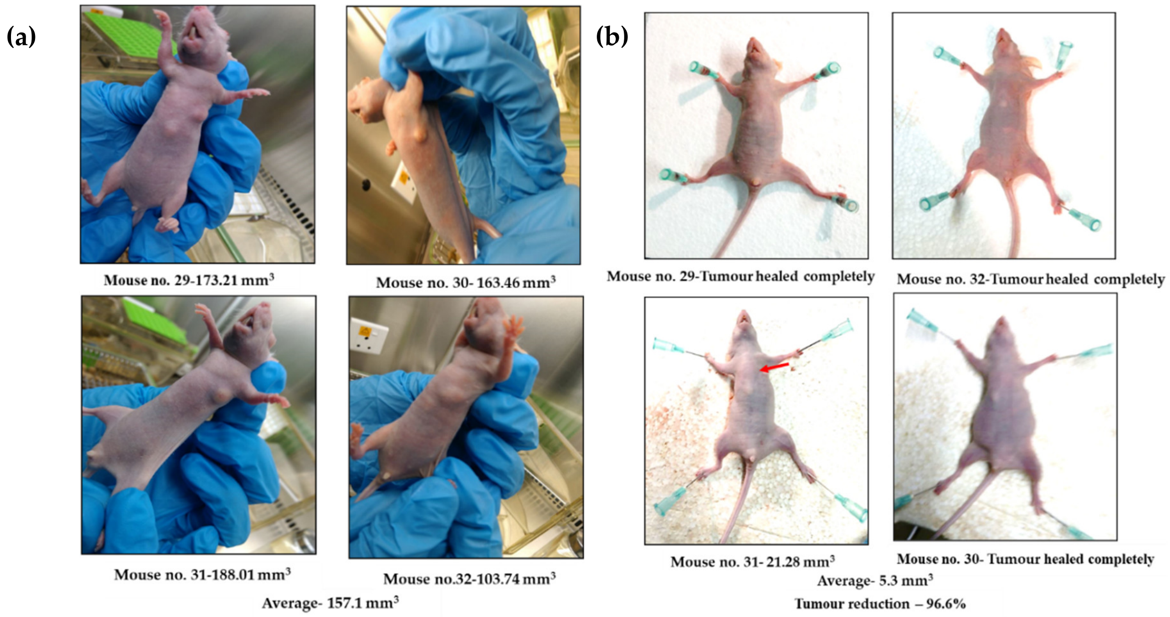

Figure S12. (**a**) Development of tumours in 4 mice (ER+). (**b**) ER+ treated with HPβCD for 5 weeks displayed a significant reduction in tumour formation following treatment with HPβCD (3000 mg/kg b.w.) leading to a 96.6% reduction of tumours in MF-1 mice relative to the untreated group.

Table S1. Case study of MDA-MB-231 H&E sections

Case	Necrosis	Epid/Muscle Invasion	Apoptosis/ 10 HPF	LVI/ PNI	Mitoses
UT 1	Present at periphery	Muscle invasion. No epidermis	17	-	59
UT 3	Focal at periphery	Extensive muscle invasion.	22	-	65 numerous atypical forms
UT 9	Present at periphery	Extensive muscle invasion. No epidermis.	27	-	57 atypical forms present
INTM 13	Focal necrosis	Muscle infiltration at the periphery.	10	-	70 atypical forms present
INTM 31	Occasional pockets of necrosis	No muscle or epidermis.	21	-	59 atypical forms present
INTM 32	Multiple pockets of necrosis	Extensive muscle invasion. No epidermis.	23	-	67 atypical forms present
LATE 8 (1)	Scattered foci of necrosis	No muscle present.	21	-	60
LATE 8 (2)	Scattered foci of necrosis	No muscle or epidermis.	30	-	65
UT 10	Scattered foci of necrosis	Muscle invasion.	22	-	77
LATE 11	Scattered foci of necrosis. Present at the periphery	Muscle invasion. Minimal uninvolved epidermis.	39	-	57
LATE 12	Necrosis seen centrally and peripherally	Muscle invasion at periphery. No epidermis present	32	-	59
LATE 14	Pockets of necrosis scattered throughout.	Muscle invasion. Epidermis is uninvolved.	27	-	50

UT – untreated, INTM – intermediate stage, LATE – late stage.
